# Increased FOXJ1 protein expression is associated with improved overall survival in high-grade serous ovarian carcinoma: an Ovarian Tumor Tissue Analysis Consortium Study

**DOI:** 10.1038/s41416-022-02014-y

**Published:** 2022-11-02

**Authors:** Ashley Weir, Eun-Young Kang, Nicola S. Meagher, Gregg S. Nelson, Prafull Ghatage, Cheng-Han Lee, Marjorie J. Riggan, Aleksandra Gentry-Maharaj, Andy Ryan, Naveena Singh, Martin Widschwendter, Jennifer Alsop, Michael S. Anglesio, Matthias W. Beckmann, Jessica Berger, Christiani Bisinotto, Jessica Boros, Alison H. Brand, James D. Brenton, Angela Brooks-Wilson, Michael E. Carney, Julie M. Cunningham, Kara L. Cushing-Haugen, Cezary Cybulski, Esther Elishaev, Ramona Erber, Sian Fereday, Anna Fischer, Luis Paz-Ares, Javier Gayarre, Blake C. Gilks, Marcel Grube, Paul R. Harnett, Holly R. Harris, Arndt Hartmann, Alexander Hein, Joy Hendley, Brenda Y. Hernandez, Sabine Heublein, Yajue Huang, Tomasz Huzarski, Anna Jakubowska, Mercedes Jimenez-Linan, Catherine J. Kennedy, Felix K. F. Kommoss, Jennifer M. Koziak, Bernhard Kraemer, Nhu D. Le, Jaime Lesnock, Jenny Lester, Jan Lubiński, Janusz Menkiszak, Britta Ney, Alexander Olawaiye, Sandra Orsulic, Ana Osorio, Luis Robles-Díaz, Matthias Ruebner, Mitul Shah, Raghwa Sharma, Yurii B. Shvetsov, Helen Steed, Aline Talhouk, Sarah E. Taylor, Nadia Traficante, Robert A. Vierkant, Chen Wang, Lynne R. Wilkens, Stacey J. Winham, Javier Benitez, Andrew Berchuck, David D. Bowtell, Francisco J. Candido dos Reis, Linda S. Cook, Anna DeFazio, AOCs group, D. Bowtell, A. DeFazio, N. Traficante, S. Fereday, A. Brand, P. Harnett, R. Sharma, Jennifer A. Doherty, Peter A. Fasching, María J. García, Ellen L. Goode, Marc T. Goodman, Jacek Gronwald, David G. Huntsman, Beth Y. Karlan, Stefan Kommoss, Francesmary Modugno, Joellen M. Schildkraut, Hans-Peter Sinn, Annette Staebler, Linda E. Kelemen, Caroline E. Ford, Usha Menon, Paul D. P. Pharoah, Martin Köbel, Susan J. Ramus

**Affiliations:** 1 School of Clinical Medicine, UNSW Medicine and Health, University of NSW Sydney, Sydney, NSW Australia; 2 Adult Cancer Program, Lowy Cancer Research Centre, University of NSW Sydney, Sydney, NSW Australia; 3 The Walter and Eliza Hall Institute of Medical Research, Parkville, VIC Australia; 4 Department of Pathology and Laboratory Medicine, University of Calgary, Foothills Medical Center, Calgary, AB Canada; 5 The Daffodil Centre, The University of Sydney, a joint venture with Cancer Council NSW, Sydney, NSW Australia; 6 Department of Oncology, Division of Gynecologic Oncology, Cumming School of Medicine, University of Calgary, Calgary, AB Canada; 7 Department of Pathology and Laboratory Medicine, University of Alberta, Edmonton, AB Canada; 8 Department of Obstetrics and Gynecology, Division of Gynecologic Oncology, Duke University Medical Center, Durham, NC USA; 9 MRC Clinical Trials Unit, Institute of Clinical Trials & Methodology, University College London, London, UK; 10 Women’s Cancer, Institute for Women’s Health, University College London, London, UK; 11 Department of Pathology, Barts Health National Health Service Trust, London, UK; 12 EUTOPS Institute, University of Innsbruck, Innsbruck, Austria; 13 Centre for Cancer Genetic Epidemiology, Department of Oncology, University of Cambridge, Cambridge, UK; 14 Department of Obstetrics and Gynecology, University of British Columbia, Vancouver, BC Canada; 15 British Columbia’s Gynecological Cancer Research Team (OVCARE), University of British Columbia, BC Cancer, and Vancouver General Hospital, Vancouver, BC Canada; 16 Department of Gynecology and Obstetrics, Comprehensive Cancer Center Erlangen-EMN, Friedrich-Alexander University Erlangen-Nuremberg, University Hospital Erlangen, Erlangen, Germany; 17 Division of Gynecologic Oncology, Department of Obstetrics, Gynecology and Reproductive Sciences, University of Pittsburgh School of Medicine, Pittsburgh, PA USA; 18 Department of Gynecology and Obstetrics, Ribeirão Preto Medical School, University of São Paulo, Ribeirão Preto, Brazil; 19 Centre for Cancer Research, The Westmead Institute for Medical Research, University of Sydney, Sydney, NSW Australia; 20 Department of Gynaecological Oncology, Westmead Hospital, Sydney, NSW Australia; 21 The University of Sydney, Sydney, NSW Australia; 22 Cancer Research UK Cambridge Institute, University of Cambridge, Cambridge, UK; 23 Canada’s Michael Smith Genome Sciences Centre, BC Cancer, Vancouver, BC Canada; 24 Department of Obstetrics and Gynecology, John A. Burns School of Medicine, University of Hawaii, Honolulu, HI USA; 25 Department of Laboratory Medicine and Pathology, Mayo Clinic, Rochester, MN USA; 26 Program in Epidemiology, Division of Public Health Sciences, Fred Hutchinson Cancer Research Center, Seattle, WA USA; 27 Department of Genetics and Pathology, International Hereditary Cancer Center, Pomeranian Medical University, Szczecin, Poland; 28 Department of Pathology, University of Pittsburgh School of Medicine, Pittsburgh, PA USA; 29 Institute of Pathology, Comprehensive Cancer Center Erlangen-EMN, Friedrich-Alexander University Erlangen-Nuremberg, University Hospital Erlangen, Erlangen, Germany; 30 Peter MacCallum Cancer Centre, Melbourne, VIC Australia; 31 Sir Peter MacCallum Department of Oncology, The University of Melbourne, Parkville, VIC Australia; 32 Institute of Pathology and Neuropathology, Tuebingen University Hospital, Tuebingen, Germany; 33 H12O-CNIO Lung Cancer Clinical Research Unit, Spanish National Cancer Research Centre (CNIO), Madrid, Spain; 34 Oncology Department, Hospital Universitario 12 de Octubre, Madrid, Spain; 35 Human Genetics Group, Spanish National Cancer Research Centre (CNIO), Madrid, Spain; 36 Department of Pathology and Laboratory Medicine, University of British Columbia, Vancouver, BC Canada; 37 Department of Women’s Health, Tuebingen University Hospital, Tuebingen, Germany; 38 Crown Princess Mary Cancer Centre, Westmead Hospital, Sydney, NSW Australia; 39 Department of Epidemiology, University of Washington, Seattle, WA USA; 40 University of Hawaii Cancer Center, Honolulu, HI USA; 41 Department of Obstetrics and Gynecology, University Hospital Heidelberg, Heidelberg, Germany; 42 Department of Genetics and Pathology, University of Zielona Gora, Zielona Gora, Poland; 43 Pomeranian Medical University, Independent Laboratory of Molecular Biology and Genetic Diagnostics, Szczecin, Poland; 44 Department of Histopathology, Addenbrooke’s Hospital, Cambridge, UK; 45 Institute of Pathology, Heidelberg University Hospital, Heidelberg, Germany; 46 Alberta Health Services-Cancer Care, Calgary, AB Canada; 47 Cancer Control Research, BC Cancer, Vancouver, BC Canada; 48 David Geffen School of Medicine, Department of Obstetrics and Gynecology, University of California at Los Angeles, Los Angeles, CA USA; 49 Department of Gynecological Surgery and Gynecological Oncology of Adults and Adolescents, Pomeranian Medical University, Szczecin, Poland; 50 Centre for Biomedical Network Research on Rare Diseases (CIBERER), Instituto de Salud Carlos III, Madrid, Spain; 51 Familial Cancer Unit and Medical Oncology Department, Hospital Universitario 12 de Octubre, Madrid, Spain; 52 Tissue Pathology and Diagnostic Oncology, Westmead Hospital, Sydney, NSW Australia; 53 Division of Gynecologic Oncology, Department of Obstetrics and Gynecology, University of Alberta, Edmonton, AB Canada; 54 Section of Gynecologic Oncology Surgery, North Zone, Alberta Health Services, Edmonton, AB Canada; 55 Department of Quantitative Health Sciences, Division of Clinical Trials and Biostatistics, Mayo Clinic, Rochester, MN USA; 56 Department of Quantitative Health Sciences, Division of Computational Biology, Mayo Clinic, Rochester, MN USA; 57 Epidemiology, School of Public Health, University of Colorado, Aurora, CO USA; 58 Community Health Sciences, University of Calgary, Calgary, AB Canada; 59 Huntsman Cancer Institute, Department of Population Health Sciences, University of Utah, Salt Lake City, UT USA; 60 Computational Oncology Group, Structural Biology Programme, Spanish National Cancer Research Centre (CNIO), Madrid, Spain; 61 Department of Quantitative Health Sciences, Division of Epidemiology, Mayo Clinic, Rochester, MN USA; 62 Cancer Prevention and Control Program, Cedars-Sinai Cancer, Cedars-Sinai Medical Center, Los Angeles, CA USA; 63 Department of Molecular Oncology, BC Cancer Research Centre, Vancouver, BC Canada; 64 Department of Epidemiology, University of Pittsburgh School of Public Health, Pittsburgh, PA USA; 65 Women’s Cancer Research Center, Magee-Womens Research Institute and Hillman Cancer Center, Pittsburgh, PA USA; 66 Department of Epidemiology, Rollins School of Public Health, Emory University, Atlanta, GA USA; 67 Hollings Cancer Center, Medical University of South Carolina, Charleston, SC USA; 68 School of Women’s and Children’s Health, Faculty of Medicine and Health, University of NSW Sydney, Sydney, NSW Australia; 69 Centre for Cancer Genetic Epidemiology, Department of Public Health and Primary Care, University of Cambridge, Cambridge, UK

**Keywords:** Tumour biomarkers, Ovarian cancer, Prognostic markers, Tumour biomarkers, Ovarian cancer

## Abstract

**Background:**

Recently, we showed a >60% difference in 5-year survival for patients with tubo-ovarian high-grade serous carcinoma (HGSC) when stratified by a 101-gene mRNA expression prognostic signature. Given the varied patient outcomes, this study aimed to translate prognostic mRNA markers into protein expression assays by immunohistochemistry and validate their survival association in HGSC.

**Methods:**

Two prognostic genes, *FOXJ1* and *GMNN*, were selected based on high-quality antibodies, correlation with protein expression and variation in immunohistochemical scores in a preliminary cohort (*n* = 134 and *n* = 80, respectively). Six thousand four hundred and thirty-four (FOXJ1) and 5470 (GMNN) formalin-fixed, paraffin-embedded ovarian neoplasms (4634 and 4185 HGSC, respectively) represented on tissue microarrays from the Ovarian Tumor Tissue Analysis consortium underwent immunohistochemical staining and scoring, then univariate and multivariate survival analysis.

**Results:**

Consistent with mRNA, FOXJ1 protein expression exhibited a linear, increasing association with improved overall survival in HGSC patients. Women with >50% expression had the most favourable outcomes (HR = 0.78, 95% CI 0.67–0.91, *p* < 0.0001). GMNN protein expression was not significantly associated with overall HSGC patient survival. However, HGSCs with >35% GMNN expression showed a trend for better outcomes, though this was not significant.

**Conclusion:**

We provide foundational evidence for the prognostic value of FOXJ1 in HGSC, validating the prior mRNA-based prognostic association by immunohistochemistry.

## Background

Ovarian carcinoma (OC) is a heterogenous disease that can be better understood through its five main histotypes being tubo-ovarian high-grade serous (HGSC), low-grade serous (LGSC), mucinous (MC), endometrioid (EC) and clear-cell (CCC) carcinoma. HGSCs are the most commonly diagnosed histotype and are typically diagnosed at a high stage. Patients diagnosed with this disease have a 5-year survival of about 40% in most Western countries [1]. Based on a prognostic signature of 101 genes, we have recently shown a dramatic difference (>60%) in median 5-year overall survival when stratified by gene expression score quintiles [2]. While the median survival time for the most favourable quintile reached 9.5 years, it was only 2.3 years in the least favourable quintile. This effect size outperforms any individual validated prognostic marker to date and further supports a remarkable biological heterogeneity within HGSC [3–6].

Historically, a blanket approach was applied to the treatment of women diagnosed with HGSC, though it is now undergoing a dramatic shift due to high response rates to poly (ADP-ribose) polymerase (PARP) inhibitors in *BRCA1/2*-deficient cases, allowing them to be added as maintenance to the first line of therapy [7, 8]. However, this targeted therapy does not show the same efficacy across all patients with HGSC, and resistance may occur[9]. Therefore, other biomarkers related to prognosis may help to further delineate the biology of this aggressive disease. Candidate biomarkers that were discovered by mRNA expression profiling could be validated by immunohistochemistry (IHC). IHC is utilised routinely in clinical diagnostics across the globe, thus supporting its use as a tool for fast-tracked, statistically powered biological validation, particularly if large existing cohorts of tissue microarrays (TMAs) are also employed [10, 11].

The purpose of this study was to translate previously identified prognostic mRNA targets into clinically applicable IHC assays and to validate their prognostic association at the protein level in HGSC and the other main OC histotypes.

## Methods

### IHC of candidate markers

Five candidate markers: geminin (*GMNN*), small nuclear ribonucleoprotein polypeptide A (*SNRPA1*), flap structure-specific endonuclease 1 (*FEN1*), histone cluster 1 H2B family member D (*HIST1H2BD*) and forkhead box J1 (*FOXJ1*), were assessed by IHC in a training cohort (*n* = 80 (GMNN, SNRPA1, FEN1 and HIST12BD) or 134 (FOXJ1). IHC was automated on the DAKO Omnis platform (Agilent, Santa Clara, CA, USA) and performed centrally at the Department of Pathology and Laboratory Medicine of the University of Calgary, Canada. TMA sections were subjected to heat-induced epitope retrieval. (Supplementary Table 1). Slides were scanned using an Aperio CS2 up to ×400 magnification (Leica Biosystems, Wetzlar, Hessen, Germany), and viewed using ImageScope v12.2.2.5015.

### Study design and participants

Sections of archival ovarian tumour tissue microarrays (TMA) were submitted by 21 studies from the Ovarian Tumor Tissue Analysis (OTTA) consortium (Supplementary Table 2) for analysis of GMNN and FOXJ1. Ethics approval was obtained for this project (University of New South Wales Human Research Ethics Advisory panel, #HC16299) and for participants through written informed consent or an Institutional Review Board ethics approval waiver (Supplementary Table 2). A retrospective cohort of 8798 and 7662 patient samples represented by individual or replicate TMA cores with a primary diagnosis of OC was assembled for FOXJ1 and GMNN staining, respectively. Contributing studies provided clinical covariates, including patient age at diagnosis, the time from diagnosis to OTTA study admission, histotype, stage of disease (FIGO (International Federation of Gynecology and Obstetrics) Stage I-IV, or localised/regional/distant), final vital status and overall survival (OS), defined as the time from diagnosis to death or last follow-up.

### Immunohistochemistry of FOXJ1 and GMNN

Positive and negative on-slide controls for FOXJ1 and GMNN protein expression were used to assess stain specificity (Supplementary Table 3). Healthy pre-menopausal and peri-menopausal fallopian tube samples (*n* = 1 case each), were also examined through haematoxylin and eosin staining, and IHC for FOXJ1 and GMNN (Supplementary Fig. 1).

IHC scoring was performed by two observers (AW and EYK). Observers were blinded to the clinicopathological data; one observer scored cases for FOXJ1 using a Nikon Eclipse 80i microscope (Nikon Inc., Chicago, IL, USA) at ×200 and another observer scored cases for GMNN using ImageScope v12.2.2.5015. Nuclear staining in each tumour core was semi-quantified by scores representing the percentage of immunopositive tumour cells in the core, in 5% intervals. Where cores were absent or tumour cells represented <25% of the core, no score was given. The maximum score was taken in cases with multiple cores on the TMAs. Intratumoural heterogeneity was assessed by a comparison of duplicate cores in *n* = 3401 (FOXJ1) and *n* = 3057 (GMNN) HGSC cases. Interobserver variability in FOXJ1 and GMNN expression scoring was evaluated in *n* = 221 and *n* = 311 cases, respectively.

### FOXJ1 and GMNN expression score stratification

IHC scores were visualised through frequency distributions of FOXJ1and GMNN scores in OC, and by histotype. Score stratification models simplified IHC scores. Thresholds used in stratification were selected to preserve the shape of distribution in the frequency histogram of HGSC scores. As FOXJ1 and GMNN expression differed in the distribution of their IHC scores, different score stratification models were applied to each data set, being FOXJ1 0%, 5%, 10–15%, 20–45% and 50–100%, and GMNN 0%, 5%, 10–15%, 20–25%, 30% and 35–100%. Score stratification was required to assess the association between expression and OS.

### Correlation analysis of mRNA and protein expression

The correlation between mRNA expression data [2] and IHC protein expression score was assessed. To ensure score stratification models would not alter this relationship, the correlation between mRNA and stratified FOXJ1 and GMNN protein was also assessed.

### FOXJ1 and GMNN protein expression in only chemotherapy-naive samples

Sensitivity univariate and multivariate survival analyses in HGSC patient where treatment by primary debulking surgery was confirmed (*n* = 4440 (FOXJ1) and n = 4009 (GMNN)) were performed. This was to ensure the effects of neoadjuvant chemotherapy (NACT) did not impact the trends detected in the main survival analysis. Paired tissue samples taken before and after NACT were also assessed for FOXJ1 and GMNN protein expression (*n* = 23 and *n* = 21 respectively) to determine whether there were changes in expression in the tissue following exposure to chemotherapy.

### Statistical analysis

The correlation between mRNA and protein expression was measured for each marker by Pearson correlation coefficients; the explainable variance was examined through the coefficient of determination. Clinical variables in FOXJ1 and GMNN cohorts were assessed through Chi-square testing of proportions. Kruskal–Wallis testing assessed IHC score differences by histotype. Squared-weighted Cohen’s kappa statistics and frequency count matrices were used to assess intratumoural heterogeneity across duplicate TMA cores from the same case. Squared-weighted Cohen’s kappa statistics were used to estimate concordance between multiple observers. A two-tailed Wilcoxon–Pratt matched-pairs signed rank test was used to assess protein expression differences in matched pairs of HGSC tumour samples taken before and after NACT.

Histotype-specific univariate and multivariate analysis of the survival associations of stratified FOXJ1 and GMNN expression were performed, with death by any cause being the primary end point. Right censoring at 10 years was applied to account for deaths not related to OC. Left truncation mitigated against survival bias introduced by time between diagnosis and enrolment into a study. Kaplan–Meier curves with corresponding log-rank testing and calculation of year-specific survival, were used to compare prognosis between strata of the score stratification model. Multivariate Cox proportional hazards regression modelling estimated hazard ratios (HRs) and corresponding 95% confidence intervals (CIs). Models were adjusted for age, stage and strata of score and stratified by OTTA study site. Assumptions of proportional hazards were tested. In cases of non-proportional covariate hazards or large sample sizes, scaled Schoenfeld residuals were plotted to assess the violation. Adjusted HRs for each strata of the score model were visualised through forest plots, and used to identify a linear relationship between expression and the hazard of death. All statistical analyses were carried out using RStudio v1.1.463 or GraphPad Prism v7.02. R packages “survival” (version 3.3-1) and “survminer” (version 0.4.9) were employed in univariate and multivariate survival analysis. Statistical significance was defined by *p* < 0.05.

## Results

### Selection of FOXJ1 and GMNN for analysis by IHC

mRNA expression analysis by NanoString was previously performed on 3769 HGSC cases from the Ovarian Tumor Tissue Analysis (OTTA) consortium and 276 genes significantly associated with patient survival were identified [2]. The 90 genes of the greatest significance were examined for suitability for IHC analysis (Supplementary Table 4). We focussed on nuclear markers (avoiding stromal or immune markers due to challenges in interpretation of staining) with high-quality antibodies suitable for IHC in formalin-fixed paraffin-embedded tissues. Five genes met these criteria: *GMNN*, *SNRPA1*, *FEN1*, *HIST1H2BD* and *FOXJ1*. The previously identified HRs for one standard deviation change in gene expression, 95% CIs and adjusted *q*-values for these genes were: *GMNN*: 0.85 (95% CI 0.82–0.89) *q* = 1.89 × 10^−11^, *SNRPA1:* 0.87 (95% CI 0.83–0.91) *q* = 1.84×10^−10^, *FEN1:* 0.89 (95% CI 0.85–0.92) *q* = 1.44 × 10^−7^, *HIST1H2BD:* 0.89 (95% CI 0.85–0.93) *q* = 1.44 × 10^−7^, *FOXJ1:* 0.90 (95% CI 0.86–0.94) *q* = 2.56 × 10^−6^ [2]. Protein expression of these markers was assessed by IHC in a small cohort of 80–134 cases. The distribution of IHC scores were examined; FOXJ1 and GMNN expression showed the greatest variation in scores (Supplementary Fig. 2). Expression of these markers was positively skewed with median expression scores of 5 and 20%, respectively. The correlation between mRNA expression data from NanoString analysis and protein expression was determined (Supplementary Fig. 2). A significant correlation between mRNA and protein expression was identified for *FEN1*, *FOXJ1* and *GMNN* (Supplementary Fig. 3). The levels of mRNA were able to explain some variance in *FOXJ1 r*^2^ = 0.283 and *GMNN r*^2^ = 0.23 protein expression. Both *FOXJ1* and *GMNN* were selected for further analysis as they showed prognostic significance at the mRNA level, demonstrated a significant correlation between mRNA and protein, and had variation in IHC scores, suggesting they could be suitable for prognostic stratification of patients.

As a marker of ciliogenesis, FOXJ1 protein expression was observed specifically in ciliated cells in pre- and peri-menopausal fallopian tube epithelium (Supplementary Fig. 1). Both pre- and peri-menopausal fallopian tube epithelium did not express GMNN (Supplementary Fig. 1). The interobserver concordance of the stratified IHC scoring systems (FOXJ1 0%, 5%, 10–15%, 20–45% and 50–100%, and GMNN 0%, 5%, 10–15%, 20–25%, 30% and 35–100%) for both FOXJ1 and GMNN expression, between 2 observers, was evaluated in a subset of 221 and 311 cases, respectively, and achieved a weighted Cohen’s kappa of 0.957 and 0.673, respectively.

### Protein expression of GMNN and FOXJ1 across OC histotypes, and the relationship with mRNA expression

**Fig. 1:**
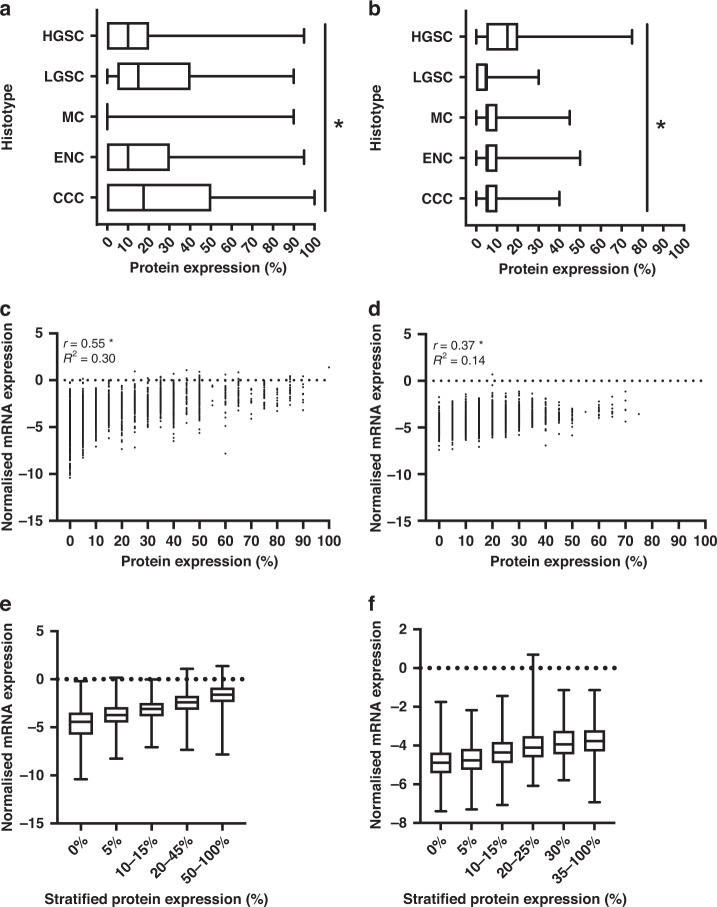
FOXJ1 and GMNN protein expression varied between histotypes, yet showed a correlation with mRNA expression in HGSC. Boxplots comparing the distribution of **a** FOXJ1 (*n* = 6434) and **b** GMNN (*n* = 5470) expression scores between histotypes. Unfilled boxes are defined by the 25th and 75th percentile, giving the interquartile range. Median scores are indicated by vertical lines within the interquartile range. If a vertical line is not shown, the median score is at the minimum or maximum value on the plot (0 or 100%, respectively). Tails indicate the minimum and maximum score given. Kruskal–Wallis testing evaluated the differences between histotypes; *p* values represent a comparison between IHC expression scores of each marker in OC histotypes. *p < 0.001. Relationship between normalised mRNA expression and protein expression in HGSC cases stained for **c** FOXJ1 (*n* = 3111) and **d** GMNN (*n* = 2900) and **e** FOXJ1 and **f** GMNN expression simplified by IHC score stratification models. Pearson’s correlation analysis given by *r* and the coefficient of determination by *R*^2^. *p < 0.0001

Of the 8798 (*FOXJ1*) and 7662 (*GMNN*) cases, 2364 and 2192 cases respectively were excluded due to diagnosis with a histotype other than HGSC, LGSC, MC, EC or CCC, no survival data, uninterpretable or insufficient tumour tissue (<25% of the tumour core), overlapping cases between TMAs or withdrawn consent. The clinicopathological parameters by histotype for 6434 FOXJ1 and 5470 GMNN cases that met the inclusion criteria were evaluated (Table 1 and Supplementary Table 5). Subsets of HGSC cases had 80% power to detect a HR of 1.3, with a type-1 error rate of 0.001. OC histotypes had significantly different FOXJ1 and GMNN expression patterns (Fig. 1). FOXJ1 protein expression varied more than GMNN expression across histotypes with greater interquartile ranges of expression scores in all histotypes (apart from MC). Intratumoural heterogeneity was assessed by weighted Cohen’s kappa statistics in duplicate HGSC TMA cores when FOXJ1 and GMNN expression were given as 5% intervals (Cohen’s kappa 0.41 and 0.40 respectively) and stratified IHC scores (Cohen’s kappa 0.74 and 0.72 respectively; Supplementary Fig. 4). 3111 *FOXJ1* and 2900 *GMNN* HGSC cases also had mRNA expression data collected through NanoString nCounter analysis [2]. The correlation between mRNA and protein expression was preserved in a HGSC subset of the final cohort to undergo survival analysis (Fig. 1). Stratification of FOXJ1 and GMNN protein expression did not appear to alter this correlation (Fig. 1).

**Table 1:** Clinicopathological characteristics by histotype of the 6434 OC patients, with complete survival data, analysed for FOXJ1 protein expression.

Characteristic	HGSC	LGSC	MC	EC	CCC
Number of cases, *n* (%)^a^	4634 (72.0)	178 (2.8)	256 (4.0)	746 (11.6)	620 (9.6)
Age at diagnosis, years					
Mean ± SD	60.6 ± 10.7	54.9 ± 12.5	53.2 ± 14.8	55.1 ± 11.7	56.2 ± 11.5
Median	61	55	53	54	56
Range	21–93	23–88	23–95	22–88	27–91
Stage, *n* (%)^b^					
FIGO I, II (localised)	846 (18.3)	54 (30.3)	216 (84.4)	625 (83.8)	473 (76.3)
FIGO III, IV (distant)	3788 (81.7)	124 (69.7)	40 (15.6)	121 (16.2)	147 (23.7)
Outcome^c^					
Alive, *n* (%)^b^	1361 (29.4)	78 (43.8)	167 (65.2)	536 (71.9)	345 (55.7)
Dead, *n* (%)^b^	3273 (70.6)	100 (56.2)	89 (34.8)	210 (28.2)	275 (44.4)
5-year survival, % ± SE	39.0 ± 0.75	59.8 ± 4.1	67.0 ± 3.2	81.3 ± 1.6	61.5 ± 2.1

*CCC* clear cell ovarian carcinoma, *EC* endometroid ovarian carcinoma, *HGSC* high-grade serous ovarian carcinoma, *FIGO* International Federation of Gynecology and Obstetrics, *LGSC* low-grade serous ovarian carcinoma, *MC* mucinous ovarian carcinoma, *SD* standard deviation, *SE* standard error.

^a^The proportion of cases in each histotypes is given as a percentage of the total patients examined.

^b^The proportion of cases is given as a percentage of the total cases within each histotypes.

^c^Final status of the patient, being alive or dead, at 10 years, following enrollment in an OTTA study.

### Association of stratified GMNN and FOXJ1 protein expression and OS, by histotype

**Fig. 2:**
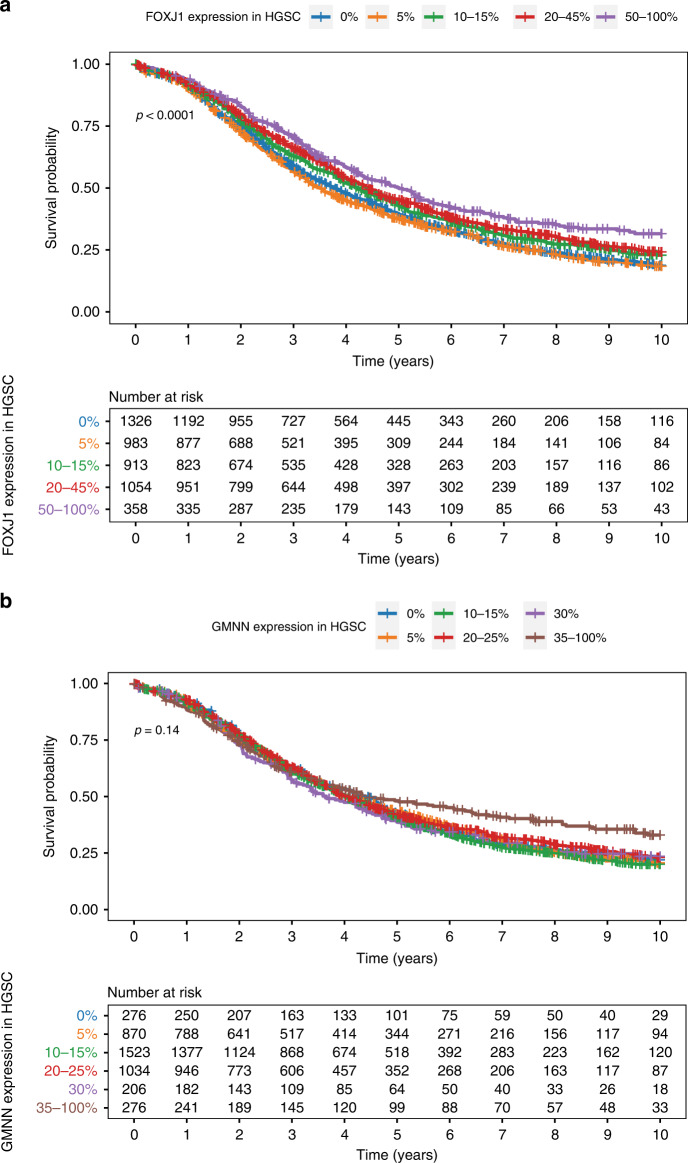
Kaplan–Meier OS curves and patients-at-risk tables. **a** OS of women with HGSC (*n* = 4634) by FOXJ1 expression status and **b** OS of women with HGSC (*n* = 4185) by GMNN expression status.

Stratified FOXJ1 protein expression was significantly associated with OS in univariate (*p* < 0.0001) and multivariate (*p* = 0.0002) analysis of HGSC survival (Fig. 2 and Table 2). Higher FOXJ1 expression was significantly associated with a higher probability of survival, relative to cases with absent or lower expression (Table 2). Prognosis was most favourable in tumours with >50% of cells expressing FOXJ1 (HR 0.78, 95% CI 0.67–0.91, *p* < 0.05), with the 5-year survival being >10% higher than those without expression.

**Table 2:** Association of stratified FOXJ1 expression and OS by histotype (*n* = 6434).

Histotype	Expression	*n*^a^	5-year survival (% ± SE)	HR (95% CI)^b^	*p* value
HGSC	0%	1336	36.0 ± 1.4	Ref.	0.0002*
	5%	987	34.1 ± 1.6	1.03 (0.94–1.14)	
	10–15%	916	40.6 ± 1.7	0.92 (0.83–1.02)	
	20–45%	1062	43.0 ± 1.6	0.86 (0.77–0.95)*	
	50–100%	358	48.0 ± 2.8	0.78 (0.67–0.91)*	
LGSC	0%	32	48.8 ± 10.36	Ref.	0.3229
	5%	24	45.9 ± 10.9	1.45 (0.63–3.35)	
	10–15%	37	59.4 ± 8.7	0.81 (0.39–1.69)	
	20–45%	50	67.7 ± 7.3	1.08 (0.53–2.21)	
	50–100%	35	66.5 ± 9.1	0.65 (0.30–1.40)	
MC	0%	216	66.5 ± 3.6	Ref.	0.2185
	5%	15	69.4 ± 12.7	9.78 (0.35–2.76)	
	10–15%	7	69.4 ± 17.9	2.24 (0.63–7.99)	
	20–45%	13	53.3 ± 15.1	1.15 (0.50–2.67)	
	50–100%	5	100.0 ± 0.0	N/A	
EC	0%	221	76.4 ± 3.1	Ref.	0.707
	5%	110	76.7 ± 4.7	0.81 (0.50–1.33)	
	10–15%	128	83.7 ± 3.8	0.76 (0.47–1.24)	
	20–45%	187	83.8 ± 2.9	0.78 (0.51–1.19)	
	50–100%	100	81.7 ± 4.0	0.94 (0.58–1.53)	
CCC	0%	174	52.9 ± 4.1	Ref.	0.03*
	5%	67	63.2 ± 6.5	0.69 (0.44–1.09)	
	10–15%	69	72.3 ± 6.1	0.50 (0.30–0.83)*	
	20–45%	147	57.0 ± 4.3	0.84 (0.60–1.18)	
	50–100%	163	69.7 ± 3.7	0.64 (0.44–0.93)*	

*CCC* clear cell ovarian carcinoma, *CI* confidence interval, *EC* endometroid ovarian carcinoma, *HGSC* high-grade serous ovarian carcinoma, *HR* hazard ratio, *LGSC* low-grade serous ovarian carcinoma, *MC* mucinous ovarian carcinoma, *OS* overall survival.

^a^The same cohort was assessed in univariate survival analysis.

^b^HR adjusted for patient age and stage and stratified by OTTA study; Cox proportional regression modelling was used to calculate *p* values and define significance. Statistically significant values are shown in bold; *p < 0.05.

Univariate and multivariate analysis of stratified FOXJ1 protein expression in CCC indicated a significant association with improved survival (*p* = 0.0096 and *p* = 0.02, respectively; Supplementary Fig. 5 and Table 2). Multivariate analysis indicated that 10–15% (HR 0.50, 95% CI 0.30–0.83, *p* < 0.05) and >50% expression (HR 0.64, 95% CI 0.44–0.93, *p* < 0.05) had a significant survival association. However, review of the Kaplan–Meier curve (Supplementary Fig. 5) did not indicate a linear relationship between FOXJ1 expression and CCC survival, whereby the probability of survival increased alongside increasing FOXJ1 expression. Similarly, no threshold effects were observed, such as any expression of FOXJ1 conferring better outcomes in comparison to tumours having no expression of the protein. No survival associations with stratified FOXJ1 expression were observed in univariate or adjusted multivariate models of LGSC, MC and EC cases (Table 2).

Stratified GMNN protein expression was not significantly associated with OS in HGSC (*p* = 0.14), though tumours with >35% GMNN expression appeared to be associated with an improved OS (Fig. 2). This trend was not supported by multivariate survival analysis adjusting for age and stage, although the overall *p* value for GMNN was significant (*p* = 0.001) (Table 3). Univariate analysis of stratified GMNN expression in EC and CCC indicated a significant association with survival (Supplementary Fig. 6), where higher expression in both cases conferred improved outcomes. However, adjusted analysis indicated that stratified GMNN expression was not significantly associated with EC or CCC patient survival (*p* = 0.09 and *p* = 0.65, respectively; Table 3). Adjusted analysis of GMNN expression in the two LGSC cases with >30% expression in tumours had significantly poorer outcomes (HR 8.28, 95% CI 1.36–50.43, *p* < 0.05; Table 3).

**Table 3:** Association of stratified GMNN expression and OS, by histotype (*n* = 5470).

Histotype	Expression	*n*^a^	5-year survival (% ± SE)	HR (95% CI)^b^	*p* value
HGSC	0%	276	38.4 ± 3.1	Ref.	0.001*
	5%	870	38.6 ± 1.7	1.10 (0.94–1.31)	
	10–15%	1523	38.3 ± 1.3	1.07 (0.91–1.26)	
	20–25%	1034	39.7 ± 1.6	0.94 (0.79–1.11)	
	30%	206	36.5 ± 3.5	1.0 (0.80–1.25)	
	35–100%	276	46.1 ± 3.2	0.80 (0.65–1.00)	
LGSC	0%	46	57.2 ± 8.3	Ref.	0.126
	5%	78	61.9 ± 6.0	0.91 (0.50–1.65)	
	10–15%	27	60.2 ± 10.3	1.23 (0.52–2.88)	
	20–25%	7	71.4 ± 17.1	2.50 (0.91–6.84)	
	30%	2	N/A	8.28 (1.36–50.43)*	
	35–100%	N/A	N/A	N/A	
MC	0%	31	77.3 ± 8.9	Ref.	0.89
	5%	67	62.8 ± 7.3	1.52 (0.61–3.80)	
	10–15%	56	74.5 ± 6.4	1.21 (0.47–3.14)	
	20–25%	18	47.9 ± 12.8	1.68 (0.58–4.86)	
	30%	1	100.0 ± 0.0	N/A	
	35–100%	1	100.0 ± 0.0	N/A	
EC	0%	80	86.0 ± 5.1	Ref.	0.1
	5%	210	87.2 ± 2.6	0.81 (0.44–1.49)	
	10–15%	162	81.8 ± 3.4	0.89 (0.47–1.68)	
	20–25%	54	62.3 ± 7.0	1.71 (0.83–3.53)	
	30%	4	25.0 ± 25.0	3.84 (0.96–15.32)	
	35–100%	8	57.1 ± 18.7	1.21 (0.24–6.09)	
CCC	0%	105	69.1 ± 5.7	Ref.	0.64
	5%	176	67.6 ± 3.8	0.94 (0.58–1.53)	
	10–15%	106	67.6 ± 4.8	0.86 (0.50–1.48)	
	20–25%	33	51.5 ± 9.2	1.10 (0.55–2.17)	
	30%	3	66.7 ± 27.2	2.62 (0.61–11.17)	
	35–100%	10	57.4 ± 16.0	0.62 (0.22–1.73)	

*CCC* clear cell ovarian carcinoma, *CI* confidence interval, *EC* endometroid ovarian carcinoma, *HGSC* high-grade serous ovarian carcinoma, *HR* hazard ratio, *LGSC* low-grade serous ovarian carcinoma, *MC* mucinous ovarian carcinoma, *OS* overall survival.

^a^The same cohort was assessed in univariate survival analysis.

^b^HR adjusted for patient age and stage, and stratified by OTTA study; Cox proportional regression modelling was used to calculate *p* values and define significance. Statistically significant values are shown in bold; *p < 0.05.

The proportional hazards assumption of Cox regression modelling was not violated by age, stage or OTTA site, in any histotypes, for both markers. To account for age and stage artefacts in these results, induced by the large sample sizes of HGSC-specific analyses, Schoenfeld residuals were plotted and assessed (Supplementary Fig. 7). No significant deviations from the line of best fit were detected.

The univariate and multivariate survival analysis results were replicated in a sensitivity analysis of a subset of the HGSC cohort, using only known chemotherapy naïve tissue, from patients where primary debulking surgery was the confirmed first line of therapy (Supplementary Fig. 8 and Supplementary Table 6).

### GMNN and not FOXJ1 protein expression was significantly different before and after NACT

FOXJ1 and GMNN protein expression in pairs of HGSC samples taken before and after NACT was assessed (Supplementary Fig. 9). While FOXJ1 expression was not significantly different in these matched pairs (*n* = 23, *p* = 0.93), GMNN expression did differ significantly after NACT (*n* = 21, *p* = 0.04).

### Internal validation of further stratified FOXJ1 protein expression in HGSC

**Fig. 3:**
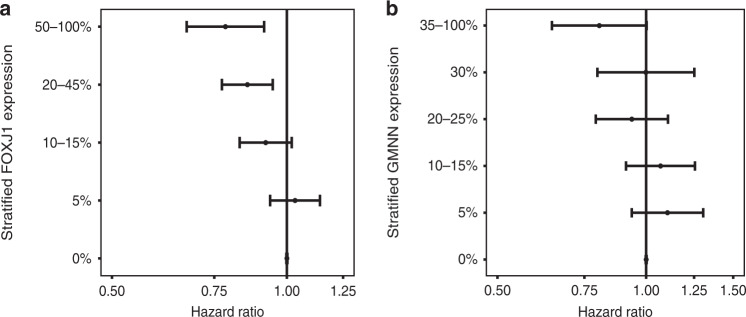
Relationship between protein expression and prognosis in HGSC. HRs adjusted for age, stage and OTTA study are shown for (**a**) FOXJ1 (*n* = 4634) and (**b**) GMNN (*n* = 4185), with standard error tails.

Forest plots were used to visualise the relationship between patient survival and FOXJ1 and GMNN score stratification models (Fig. 3). A linear relationship between FOXJ1 protein expression and the risk of death was observed in HGSC patients (Fig. 3). As FOXJ1 expression increased the probability of HGSC patient survival also increased. The HR for GMNN expression was not linear but was suggestive of a threshold-effect (Fig. 3). Cases with >35% of tumour cells expressing GMNN were less likely to succumb to disease, than those with lower expression.

## Discussion

Our study validates the linear, increasing prognostic association of FOXJ1 protein expression in HGSC in a large, international cohort. While our analysis of FOXJ1 exemplifies the power of IHC for rapid biological validation of prognostic biomarkers that were originally discovered as mRNA candidates, the lack of translation of other markers also illustrates the challenges.

As FOXJ1 protein expression increased, the probability of survival in HGSC increased, implying expression has a linear association with outcome. Patients with tumours with >50% of cells expressing FOXJ1 had the longest survival. Sui and colleagues have already suggested that high FOXJ1 expression is associated with favourable OC prognosis [12]. The present analysis uses a statistically powered, histotype-specific approach to differentiate that FOXJ1 is only associated with prognosis in HGSC and does not indicate the prognosis of other histotypes, perhaps except for CCC. High expressing CCC were associated with favourable survival as were cases with lower expression, contrasting the observed linear association with survival in HGSC. Sui and colleagues proposed that the stem cell-related transcription factor NANOG is a negative regulator of FOXJ1-mediated OC migration and invasion [12]. This suggests that NANOG high/FOXJ1 low are more akin to cancer stem cells, which may be more likely chemo-resistant and thereby less susceptible to chemotherapy, leading to poor prognosis. However, we were unable to address the response to chemotherapy in the current data set due to lack of data, e.g. the Response Evaluation Criteria in Solid Tumors (RECIST) data. Yet this generates an interesting hypothesis for future studies.

The biological significance and prognostic association of FOXJ1 in cancer is varied [13, 14]. Survival associations consistent with our HGSC results have been observed in gastric cancer, as well as ependymomas and choroid plexus tumours, where high expression of FOXJ1 is thought to be a marker of better tumour differentiation and a more favourable prognosis [15, 16]. It may be appealing to conclude that FOXJ1 is a marker for well-differentiated HGSCs. FOXJ1’s characteristic role is in motile ciliogenesis, and we confirmed FOXJ1 expression in ciliated, but not secretory cells of the fallopian tube. However, this would somewhat contradict the prevailing concept that HGSC arises from the secretory cells, as opposed to ciliated cells, of the fallopian tube via precursor serous tubal intraepithelial carcinoma (STIC) lesions and diffuse PAX8 staining, a marker of secretory cells, is present in virtually all HGSCs, supporting the STIC hypothesis [17–21]. As motile cilia are neither a feature of STIC nor HGSC, the expression of FOXJ1 does not suggest that HGSC arise from terminally differentiated ciliated cells of the fallopian tube or that a cell lineage switch occurs [22]. Instead, the expression of the ciliated marker FOXJ1 in HGSC can be considered as a marker of enigmatic differentiation, which does not result in morphologically apparent cilia and is associated with better outcome; a phenomenon that has been previously suggested for endometrial cancers [23]. “Aberrant” FOXJ1 expression in HGSC may reflect epigenetic reprogramming of secretory cell-derived tumour cells or a unique differentiation state of the cell of origin. A recent study proposed that HGSC are composed of a mixture of cancer cell states inherited from its cell of origin in the fallopian tube including a ciliated state [24]. An enigmatic ciliated differentiation is further supported by expression of other ciliated markers (such as Ezrin) in OC confirming that FOXJ1 expression is not an isolated, aberrantly expressed marker of ciliogenesis, but represents a more general differentiation programme [25, 26]. In addition to FOXJ1’s potential in HGSC prognostication, future studies may use FOXJ1 to gain further insights into HGSC development.

Contrary to results in FOXJ1, the univariate survival analysis with GMNN expression indicated that there was no significant prognostic association in HGSC. Contrastingly, multivariate survival analysis did indicate that GMNN expression was significantly associated with HGSC survival, although there were no observable trends in HRs across protein expression groups that suggested this association was linear. In a threshold effect, individuals with >35% of tumour tissue expressing GMNN did demonstrate a trend towards a more favourable prognosis, although this was not supported by the 95%CI in the multivariate analysis. As *GMNN*’s significant mRNA association with survival did not translate into protein expression, GMNN is not currently suitable for prognostic assessment through IHC. Various explanations of this result can be drawn. Large-scale studies have shown the correlation between mRNA and protein expression is varied and difficult to predict for a given biomarker [27, 28]. In the final cohort, the correlation between mRNA and GMNN protein expression was weaker, relative to FOXJ1 expression. While this difference was slight, it may have contributed to the result. Dissimilar mRNA and protein abundances may be reflective of the translation rate, its modulation, delays in protein synthesis and transport, and the modulation of the proteins’ turnover-rate [29–31]. As a surrogate marker of proliferation, GMNN’s expression is highly regulated during the cell cycle [32]. Protein translation and degradation may be uncoupled from mRNA transcription and stability, and may explain the lack of association with survival for GMNN protein levels. Another explanation might be that the FOXJ1 IHC assay was superior in quality and therefore easier to interpret as shown by the better interobserver reproducibility. Perhaps another reason that GMNN did not translate into a suitable prognostic IHC marker is the less variable expression within HGSC, when compared to FOXJ1. While prognostic significance was not shown in LGSC, multivariate analysis indicated that individuals with >35% of tumour tissue expressing GMNN were associated with a more unfavourable prognosis. However, as this group only included two individuals this should only be considered an interesting observation highlighting the challenges of biomarker studies in rare histotypes even in a consortium type approach. No associations were seen in the other histotypes. GMNN expression did however differ significantly in samples taken before and after NACT in small cohort of HGSC cases. This difference can potentially be attributed to the role of GMNN in cell division alongside the impact of chemotherapy on this process [33]. However, trends that would suggest GMNN expression increased or decreased following NACT were not observed. Furthermore, the potential clinical relevance of this result is limited by the small sample size, which would not completely capture disease heterogeneity. HGSC cases known to be chemotherapy naïve underwent univariate and multivariate survival analysis to confirm the prognostic associations revealed in this study.

The potential for FOXJ1 and GMNN expression stratification into broader score-groupings was observed in our HGSC cohort. As 0% and 5%, as well as 10%-15% and 20%-45% exhibited consistently similar survival associations in the cohort, analysis of FOXJ1 expression by 0%-5%, 10-45% and 50%-100% IHC score groupings may prove more-appropriate for clinical assessment. Similarly, the trend observed in tumours with high GMNN expression suggested that stratification into 0%-35% and 40%-100% may clarify the relationship with survival, though the lack of significant results in this study, does render the value of further GMNN investigation dubious. We acknowledge that the translational potential of our findings is limited by heterogeneity within HGSC. Future work may take important molecular alterations within HGSC, such *BRCA1/2* mutation or homologous repair deficiency status, into account. Further, we recognise that the immediate translational potential of FOXJ1 as an individual prognostic biomarker is limited given the observed survival increase of ~10% in a small subset (less than 10%) of HGSC patients. Yet we believe that due to robust prognostic associations FOXJ1 should be evaluated in prognostic multimarker models.

Several variables can interfere with the translation of mRNA targets into IHC biomarkers. Our marker selection was severely limited by the number of genes with available high-quality monoclonal antibodies, which were necessary as they ensured increased stain reproducibility across FFPE TMA sections. We avoided proteins of the microenvironment and focussed on those with nuclear expression as they are often the most robust markers for established clinical tests. When selecting markers, we observed only moderate correlations between mRNA and protein expression for the five initially selected markers. While gene expression or protein stability can influence these correlations, we believe that a main factor is the use of conventional IHC, which is not quantitative in nature [34–37]. Modern IHC detection systems greatly amplify the signal leading to a quick saturation. This might explain why some of the initial markers showed ubiquitous expression by IHC in comparison to variable mRNA expression levels. In addition, markers were assessed on TMAs with an intrinsic limitation in assessing for intratumoural heterogeneity. However, our assessment of intratumoural heterogeneity in cases with duplicate TMA cores demonstrated strong agreement in FOXJ1 and GMNN protein expression scores. While this may not capture all variation in protein expression across each tumour, it does support that the scores used in our analysis were representative of the tumour profile.

Overall, our study has provided foundational evidence that the prognostic mRNA signature of *FOXJ1* can be translated into an IHC biomarker, to stratify prognosis in HGSC. Survival improved with stratified, increasing FOXJ1 protein expression. It indicated this relationship could be further stratified, to improve clinical utility, which is intriguing regarding its unexpected expression in HGSC. We also demonstrate the challenges of validating prognostic IHC markers. The careful study design and power has ensured these results provide much-needed insights into the likely outcomes of HGSC patients.

## Supplementary information

Checklist

Supplementary Figures

Supplementary Tables 1, 3, 4, 5 and 6

Supplementary Table 2

AOCS Study Group

## Data Availability

Participants of this study did not agree to their data being shared publicly; accordingly, the data used in this research will not be made available.
