# Supplementary material for: Increased FOXJ1 protein expression is associated with improved overall survival in high-grade serous ovarian carcinoma: an Ovarian Tumor Tissue Analysis Consortium Study
Source: Br J Cancer. 2022 Nov 2;128(1):137–47. doi: 10.1038/s41416-022-02014-y (PMC9814937; doi:10.1038/s41416-022-02014-y)
Supplement: Supplementary file 2 — Supplementary Figures [file 41416_2022_2014_MOESM2_ESM.docx]

**Increased FOXJ1 protein expression is associated with improved overall survival in high-grade serous ovarian carcinoma: an Ovarian Tumor Tissue Analysis Consortium Study**

**Supplementary figures**

| **A** | **B** |
| --- | --- |
| **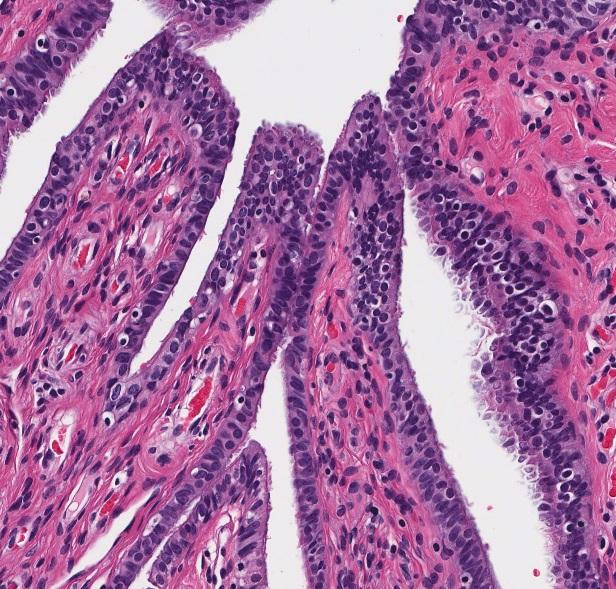** | **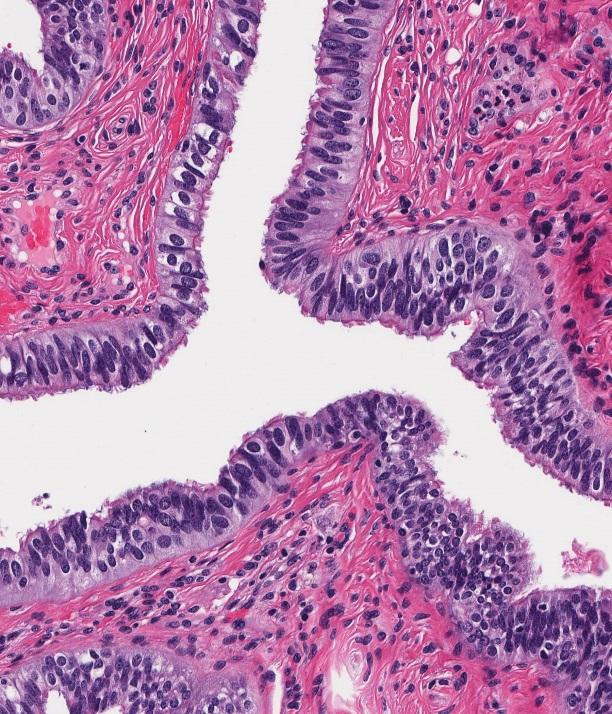** |
| **C** | **D** |
| **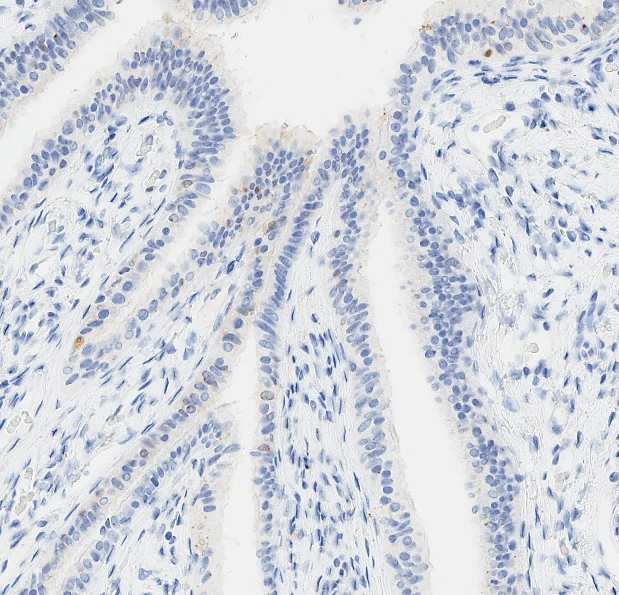** | **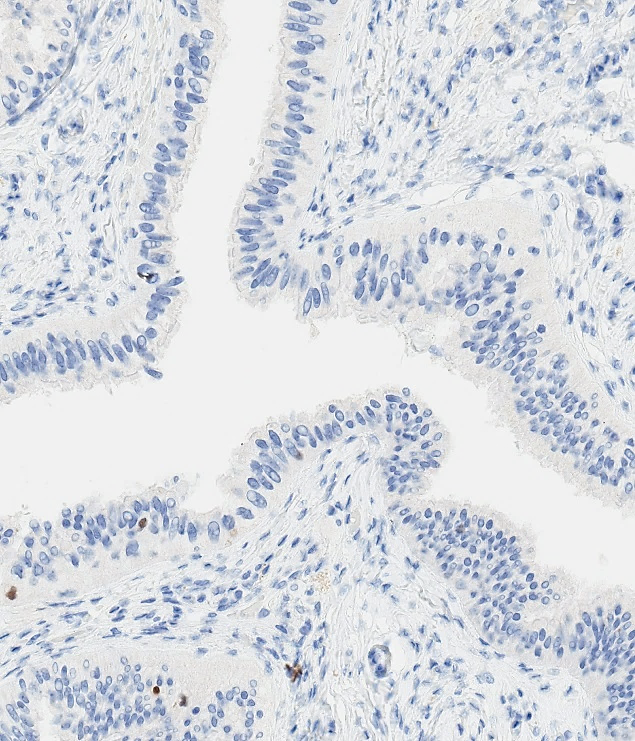** |
| **E** | **F** |
| **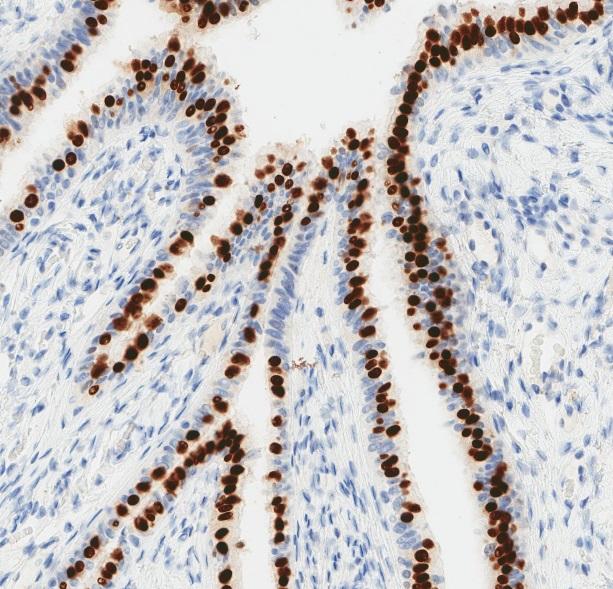** | **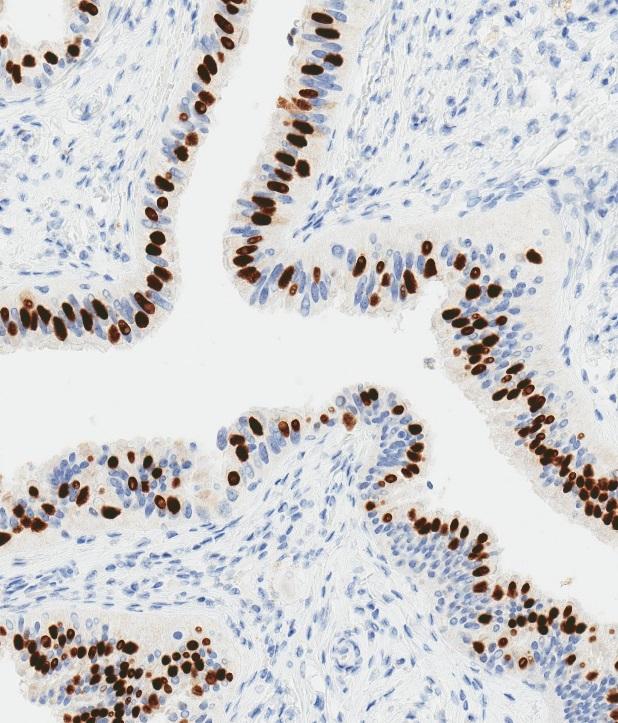** |
|  |  |
| **Supplementary figure 1** Healthy fallopian tube from a (A), (C), (E) premenopausal woman and a (B), (D), (F) perimenopausal woman. (A), (B) Hematoxylin and eosin staining of tissue. IHC staining for (C), (D) GMNN and (E), (F) FOXJ1 expression. | |

| **A** | **B** |
| --- | --- |
| **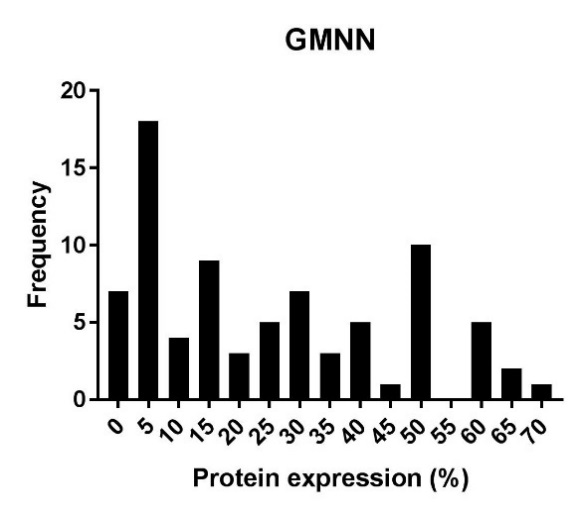** | 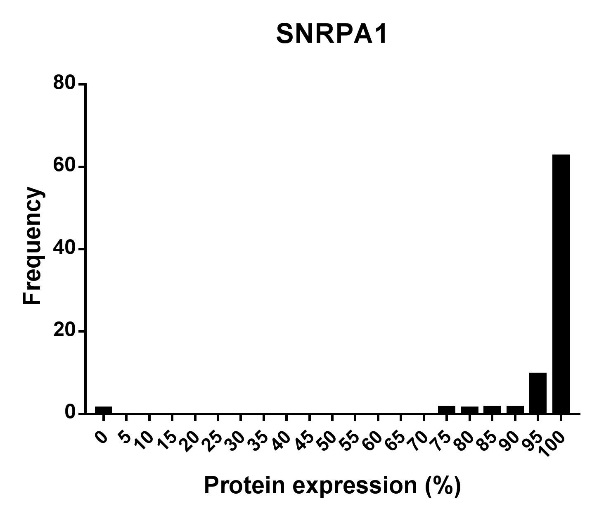 |
| **C** | **D** |
| 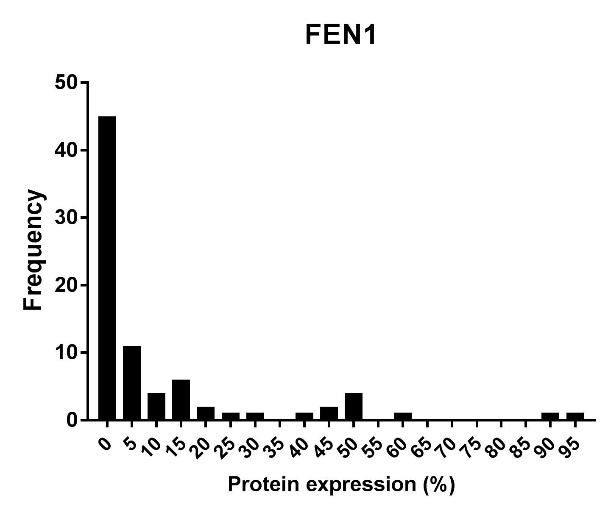 | 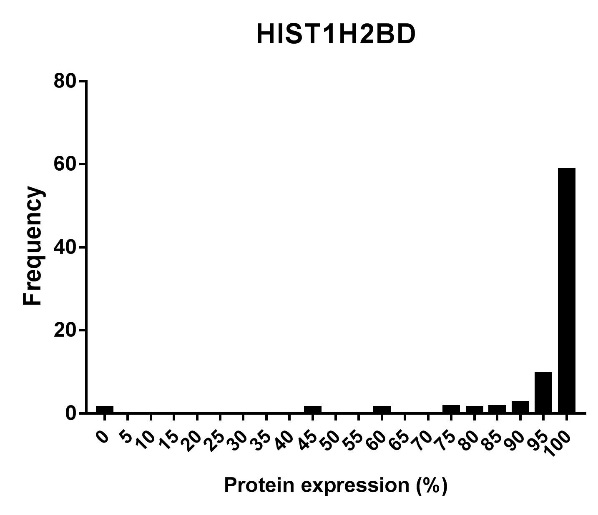 |
| **E** | **F** |
| **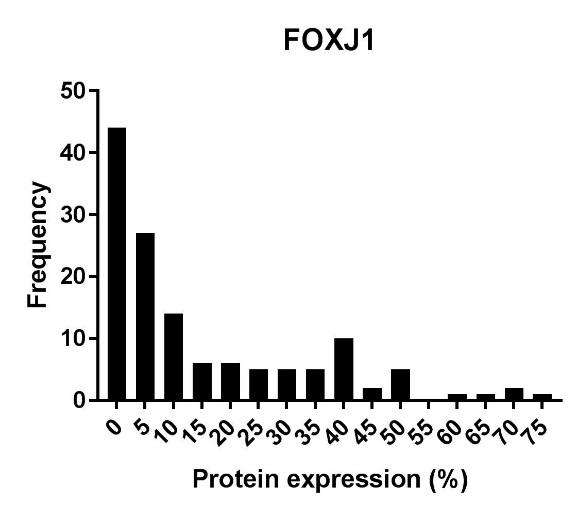** | 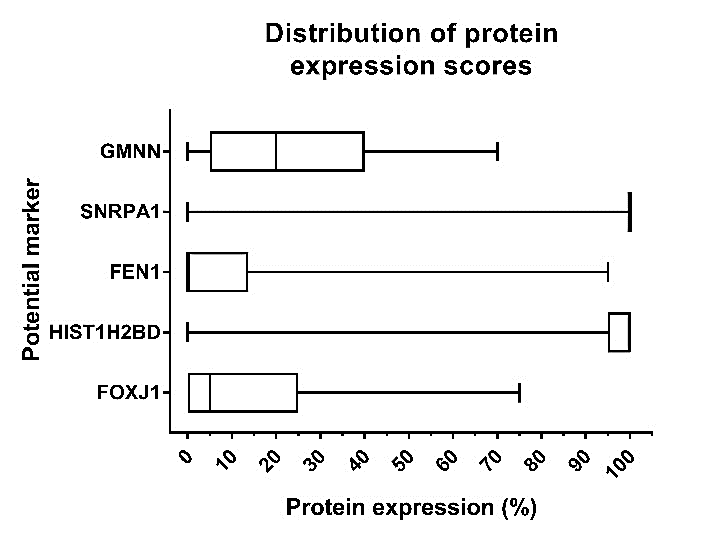 |

| **Supplementary figure 2** Summary of protein expression by immunohistochemistry scores for potential biomarkers. Frequency distribution of protein expression scores in a subset of n=80 cases stained for (A) GMNN, (B) SNRPA1, (C) FEN1 and (D) HIST1H2BD, and n=134 cases for (E) FOXJ1. (F) Boxplots comparing the distribution of expression scores for each marker. Unfilled boxes are defined by the 25^th^ and 75^th^ percentile, giving the interquartile range. Median scores are indicated by vertical lines within the interquartile range. If a vertical line is not shown, the median score is at the minimum or maximum value on the plot (0% or 100% respectively). Tails indicate the minimum and maximum score given. |
| --- |

| **A** | **B** |
| --- | --- |
| **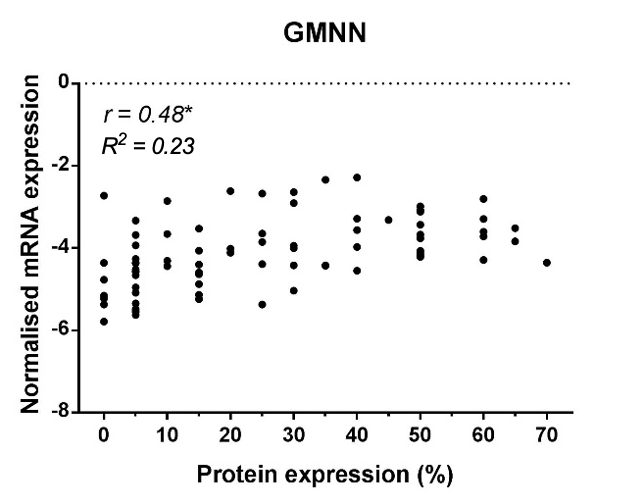** | **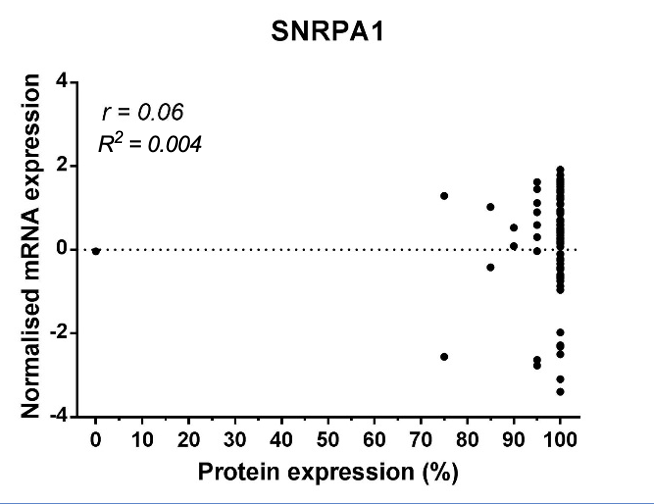** |
| **C** | **D** |
| **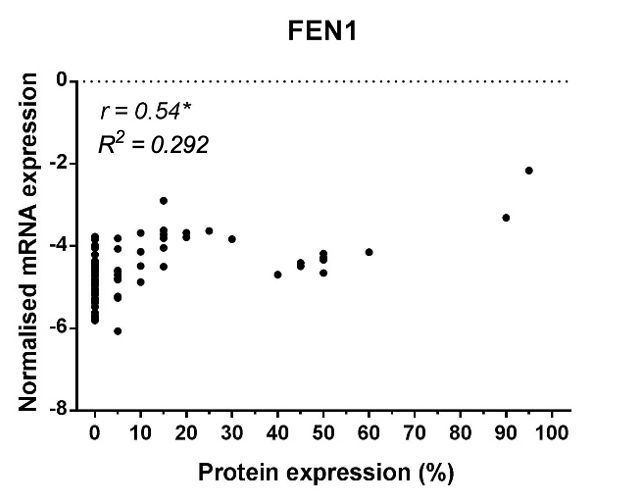** | **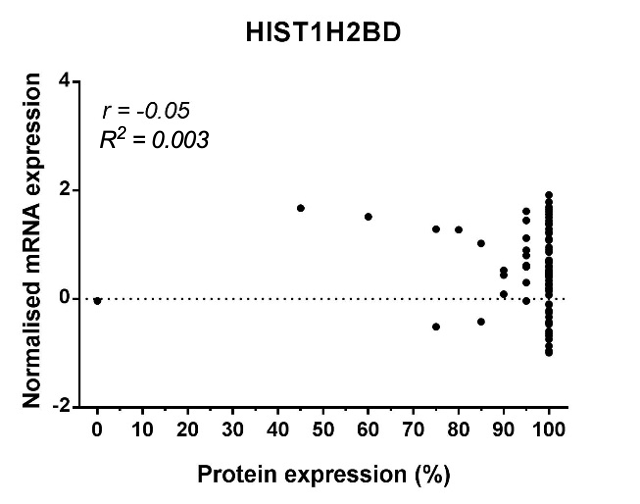** |
| **E** |  |
| **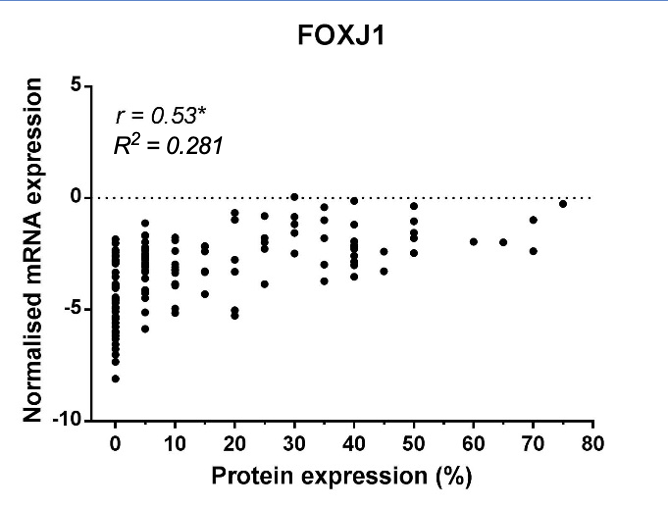** |  |
|  |  |
| **Supplementary figure 3** Correlation between normalised mRNA expression and protein expression in n=80 OC cases stained for (A) GMNN, (B) SNRPA1, (C) FEN1 and (D) HIST1H2BD, and n=134 OC cases for (E) FOXJ1. Pearson’s correlation analysis given by r and the coefficient of determination by $R^{2}.$* p<0.0001 | |

| **A** | **B** |
| --- | --- |
|  |  |
| **C** | **D** |
|  |  |
|  |  |
| **Supplementary figure 4** Frequency count matrices comparing IHC scores across two TMA cores of the same HGSC case. Frequency counts of IHC scores in 5% intervals of (A) FOXJ1 (n=3401) and (B) GMNN (n=3057) expression or in the same cohorts, stratified as (C) FOXJ1 expression at 0%, 5%, 10-15%, 20-45% and 50-100% and (D) GMNN expression at 0%, 5%, 10-15%, 20-25%, 30% and 35-100%. | |

| **A** |
| --- |
| 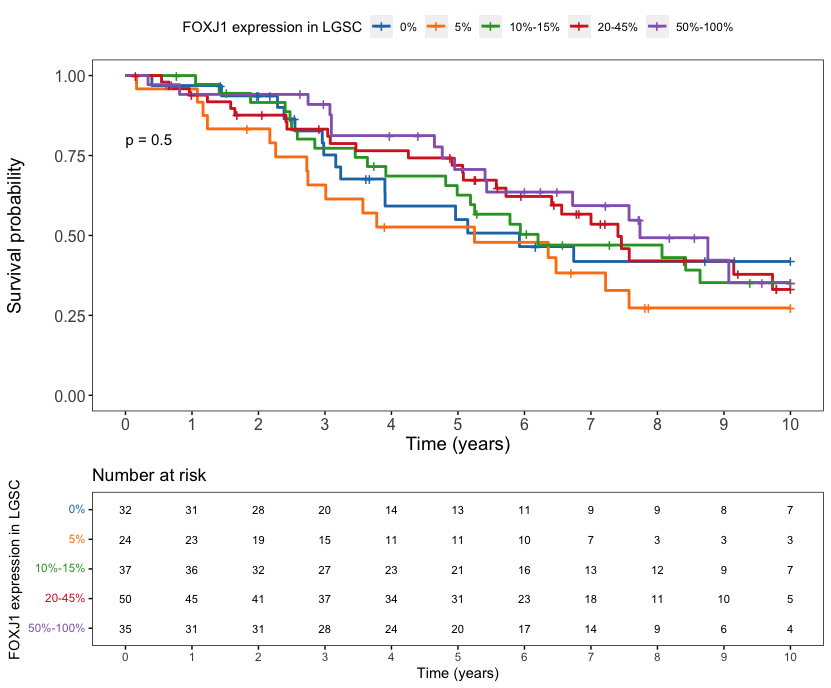 |
| **B** |
| 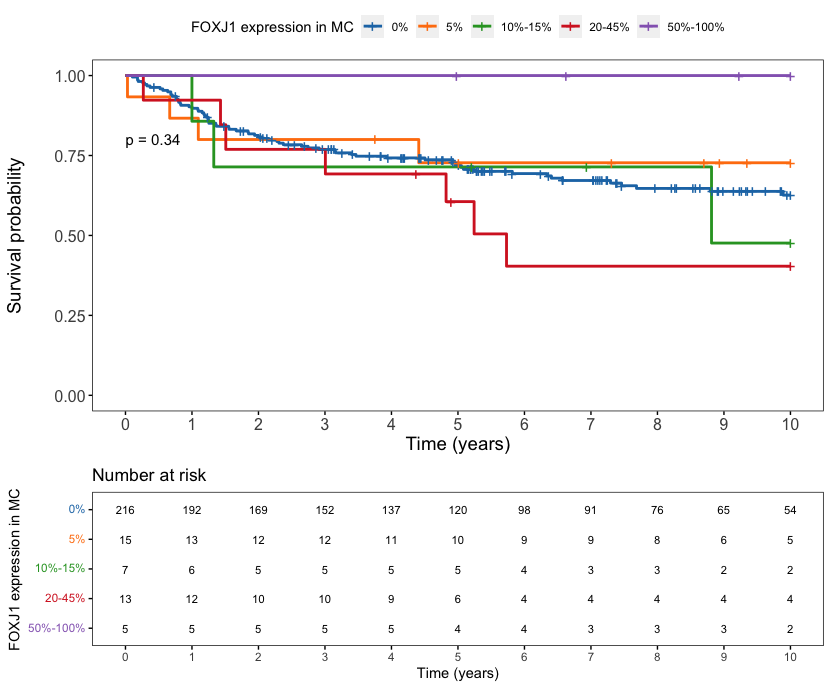 |
| **C** |
| 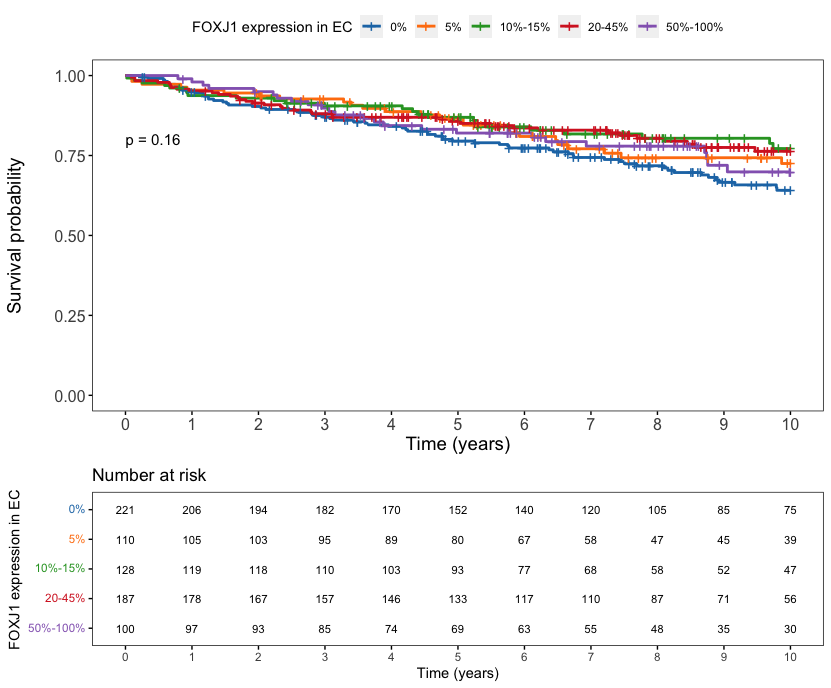 |
| **D** |
| 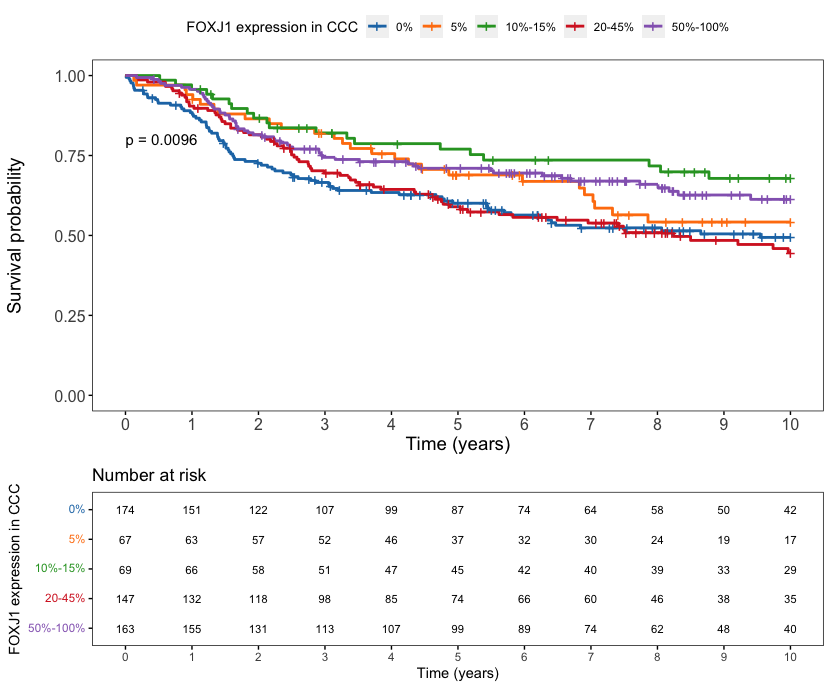 |
|  |
| **Supplementary figure 5** Kaplan-Meier OS curves and patients-at-risk tables of FOXJ1 expression, evaluated by IHC, in women with (A) LGSC (n=178), (B) MC (n=256), (C) EC (n=746) and (D) CCC (n=620). p-values calculated through log-rank testing. |

| **A** |
| --- |
| 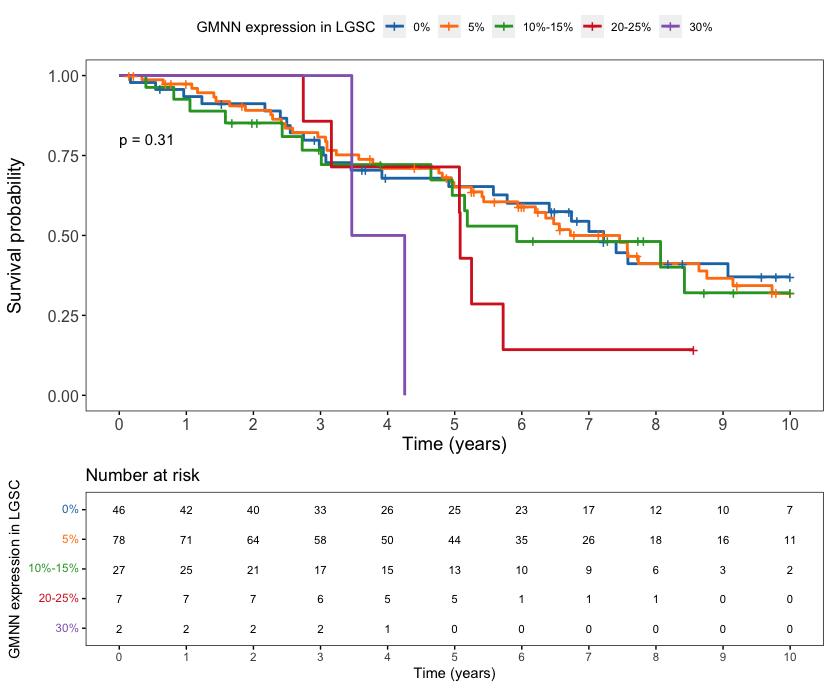 |
| **B** |
| 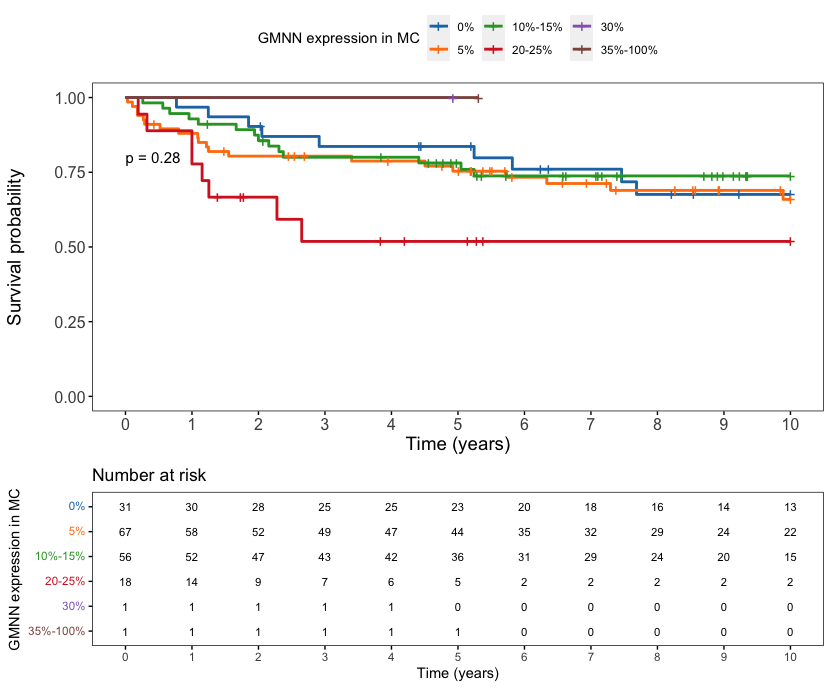 |
| **C** |
| 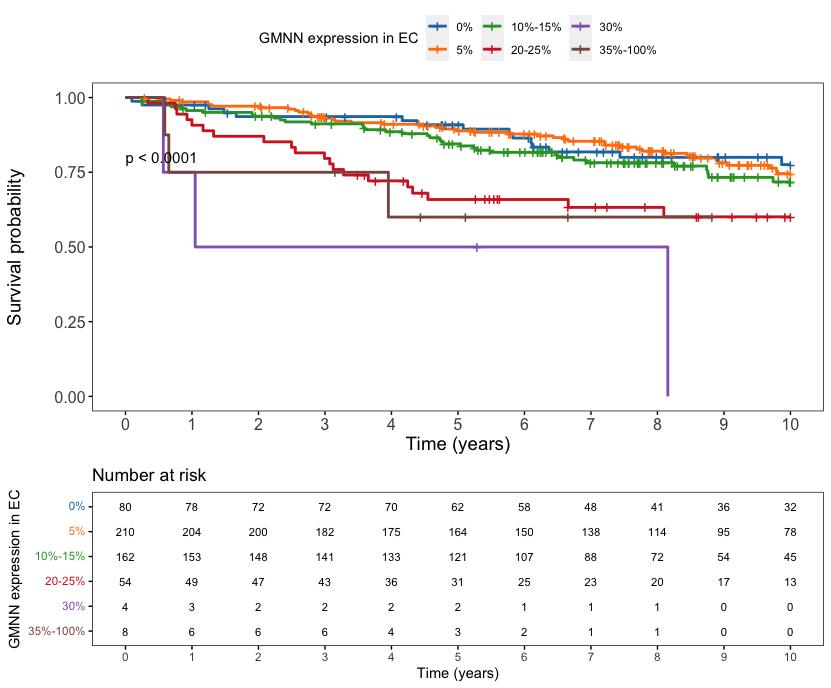 |
| **D** |
| 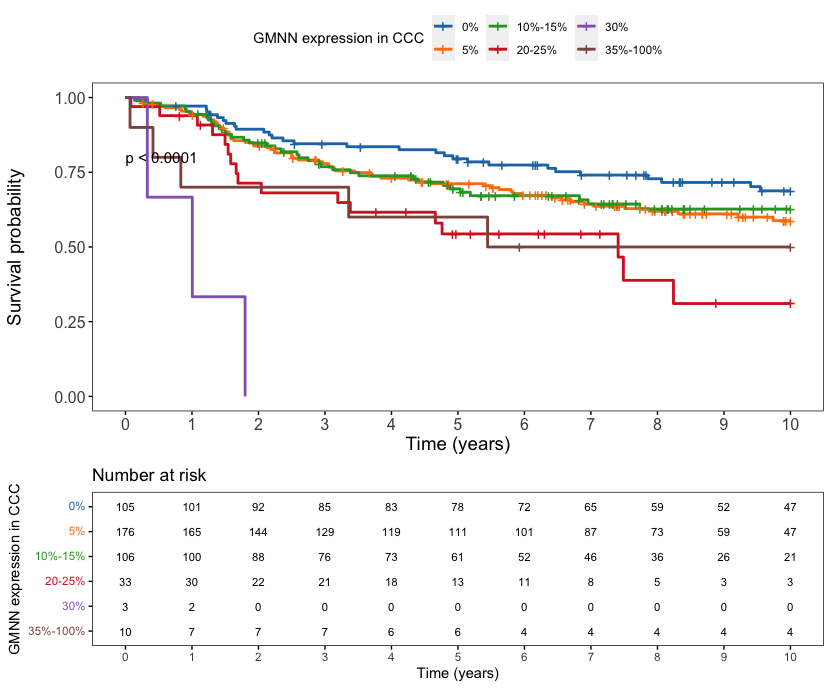 |
|  |
| **Supplementary figure 6** Kaplan-Meier OS curves and patients-at-risk tables of GMNN expression, evaluated by IHC, in women with (A) LGSC (n=160), (B) MC (n=174), (C) EC (n=518) and (D) CCC (n=433). p-values calculated through log-rank testing. |

| **A** | **B** |
| --- | --- |
| **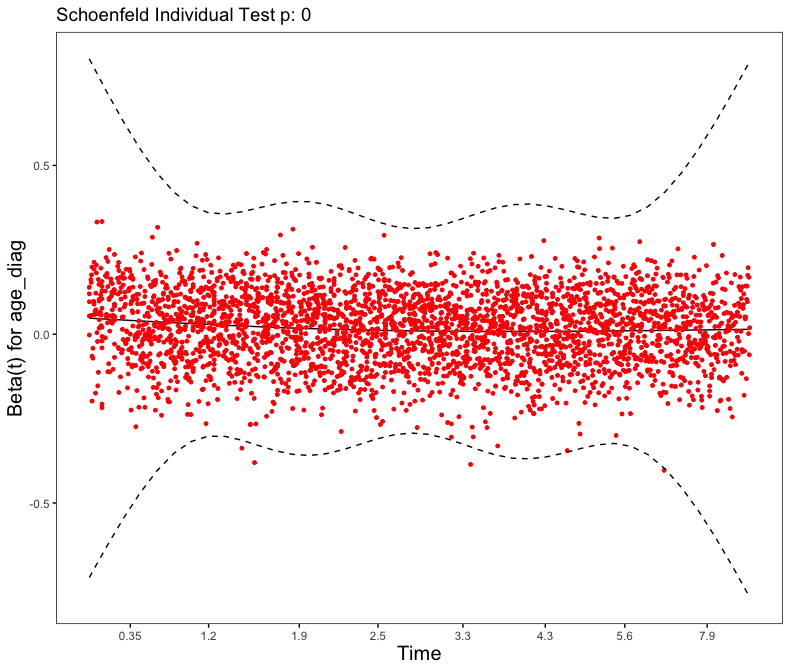** | **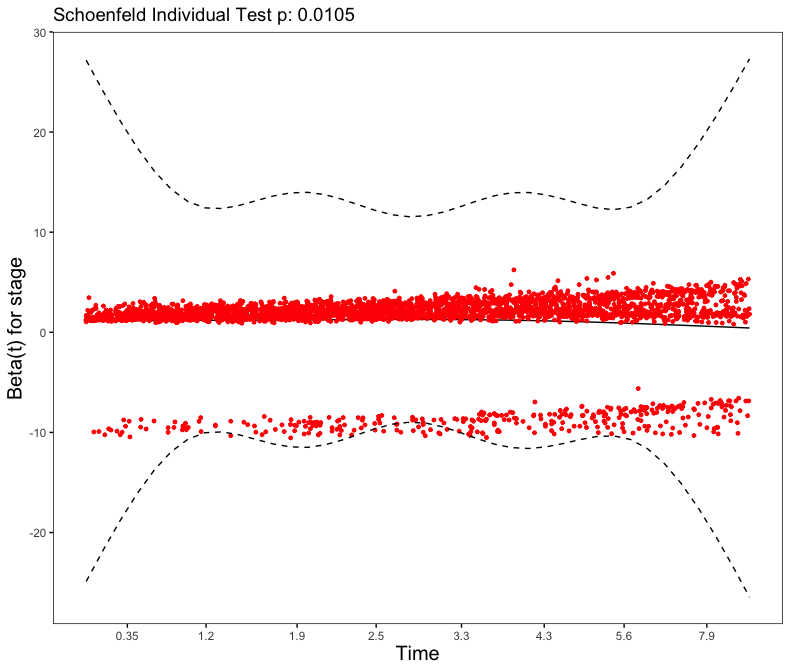** |
| **C** | **D** |
| **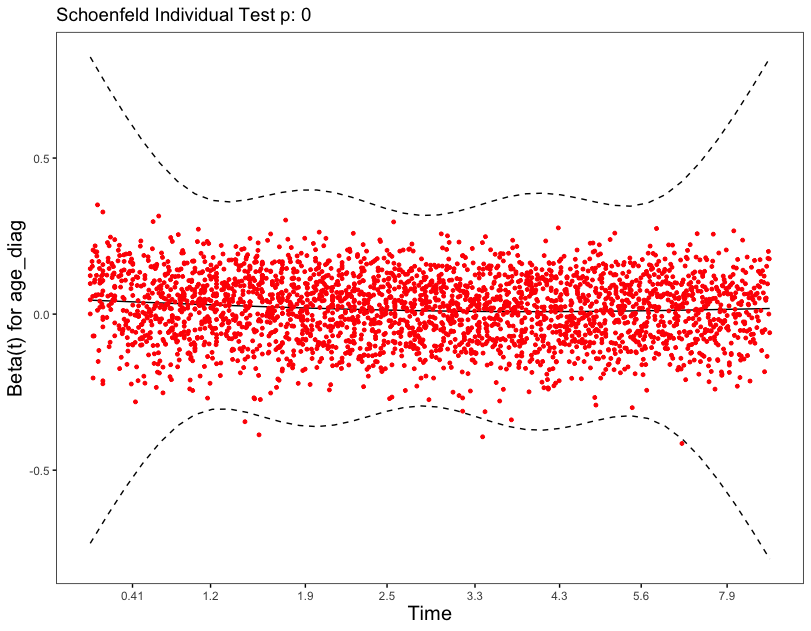** | **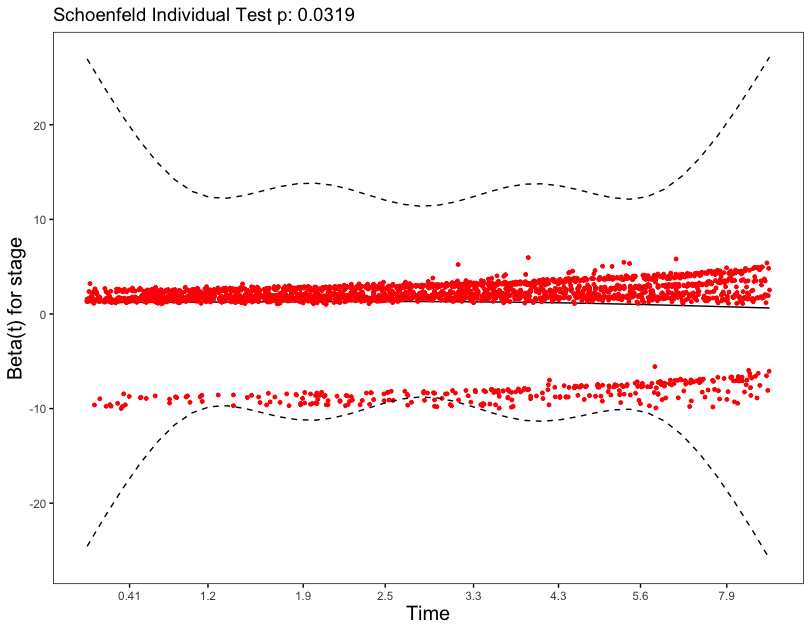** |
| **Supplementary figure 7** Scaled Schoenfeld residuals plotted to against transformed time, to assess potential time-dependent interactions of (A) age at diagnosis and (B) stage in HGSC cases expressing FOXJ1 (n=4634), and (C) age at diagnosis and (D) stage in HGSC cases expressing GMNN (n=4185). Smoothing splines approximate the time-dependency of the coefficients and are given by solid lines, with corresponding ±2-standard error bands given by dashed lines. Scaled beta residuals are given by red points spread about the smoothing spline. Schoenfeld individual testing p-value indicates the significance in the models’ goodness of fit, however due to the large sample size, it is less reliable than the spread of residuals about the smoothing spline in signifying a time-dependent covariate. | |

| **A** |
| --- |
| **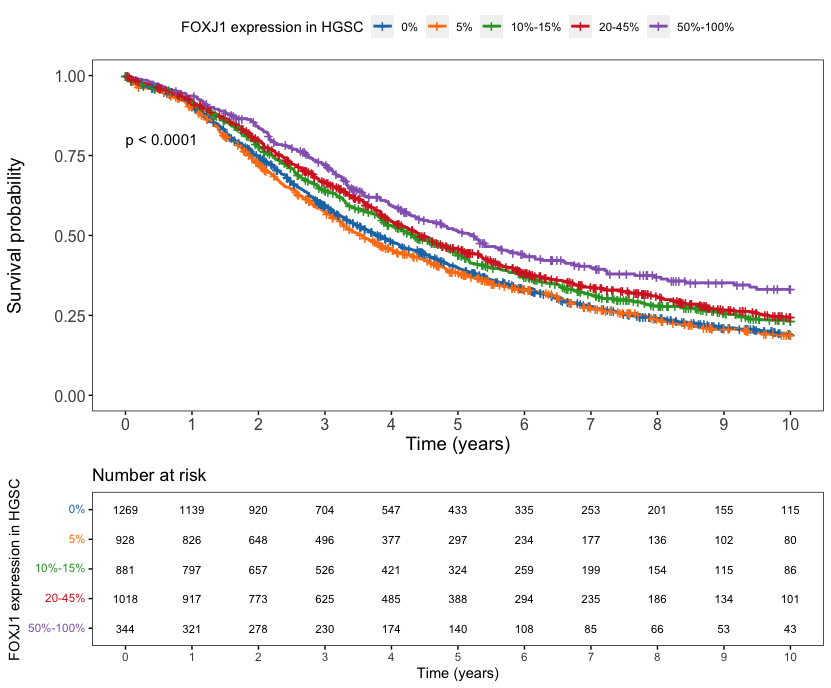** |
| **B** |
| **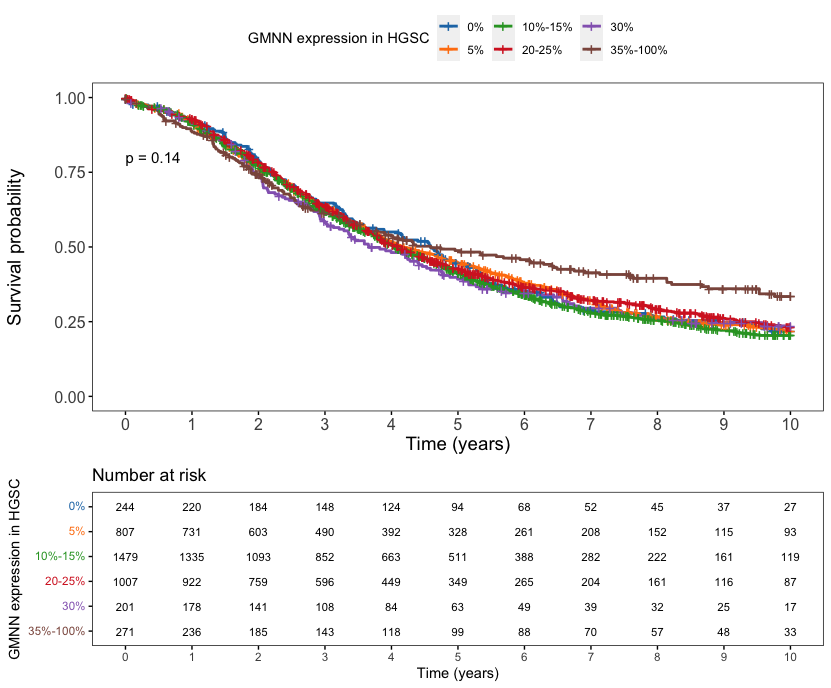** |
|  |
|  |
| **Supplementary figure 8** Kaplan-Meier OS curves and patients-at-risk tables of (A) FOXJ1 and (B) GMNN protein expression in HGSC cases (n=4440 and n=4009 respectively) where primary debulking surgery was the first line of therapy. p-values calculated through log-rank testing. |

| **A** | **** | **B** | **** |
| --- | --- | --- | --- |
|  |  |  |  |
| **C** | **** | **D** | **** |
|  | | | |
| **Supplementary figure 9** Matched pairs of tumour samples collected before and after NACT, stained for FOXJ1 (A,C) and GMNN (B,D) protein expression in HGSC (n=23 and n=21 pairs). Dots represent immunohistochemistry scores given as 5% intervals in protein expression before and after NACT (A.B). Track lines connect protein expression scores belonging to the same sample. Boxplots comparing the distribution of (C) FOXJ1 and (D) GMNN expression scores when samples were taken before and after NACT. Unfilled boxes are defined by the 25^th^ and 75^th^ percentile, giving the interquartile range. Median scores are indicated by vertical lines within the interquartile range. Tails indicate the minimum and maximum score given. | | | |
