# Supplementary material for: Increased FOXJ1 protein expression is associated with improved overall survival in high-grade serous ovarian carcinoma: an Ovarian Tumor Tissue Analysis Consortium Study
Source: Br J Cancer. 2022 Nov 2;128(1):137–47. doi: 10.1038/s41416-022-02014-y (PMC9814937; doi:10.1038/s41416-022-02014-y)
Supplement: Supplementary file 3 — Supplementary Tables 1, 3, 4, 5 and 6 [file 41416_2022_2014_MOESM3_ESM.pdf]

Increased FOXJ1 protein expression is associated with improved overall survival in high-grade serous ovarian carcinoma: an Ovarian Tumor Tissue Analysis Consortium Study

Supplementary material

Supplementary table 1 Monoclonal primary antibodies used in IHC staining of OC tumours in TMAs, contributed to this project by OTTA studies

Supplementary table 2 OTTA studies participating in the study and ethics approval **(uploaded separately)**

Supplementary table 3 On-slide positive and negative tissue controls for GMNN and FOXJ1 IHC staining, arrayed on TMAs examined in the final scoring cohorts of 5503 and 7759 OC cases, respectively

Supplementary table 4 Top 90 prognostic genes identified by NanoString analysis, ranked by their association with HGSC survival (n=3,769)

Supplementary table 5 Clinicopathological characteristics by histotype of the 5470 OC patients, with complete survival data, examined by GMNN expression

Supplementary table 6 Association of stratified FOXJ1 and GMNN protein expression and OS in HGSC, where cases were known to have not been treated with NACT (n=4440 and 4009 respectively)

Supplementary table 1 Monoclonal primary antibodies used in IHC staining of OC tumours in TMAs, contributed to this project by OTTA studies

Protein of interest	Antibody [clone] (catalogue number)[a]	Epitope recognised by the antibody[b]	Dilution	Linker used to amplify signal[c]	Recommended positive IHC controls[d]
GMNN	Anti-Geminin antibody [EPR14637] (ab195047)	Geminin AA 100 to the C-terminus	1/1000	N/A[e]	Tonsil
FOXJ1	Anti-FoxJ1 antibody [EPR21874] (ab235445)	FOXJ1 AA 50 to the C-terminus	1/300	EnVision FLEX+ rabbit linker	Normal fallopian tube tissue
FEN1	Anti-FEN1 antibody [EPR4460(2)] (ab109132)	FEN1 AA 300-400	1/500	N/A[e]	Tonsil, intestinal epithelium
HIST1H2BD	Anti-Histone H2B (acetyl K20) antibody [EPR859] (ab177430)	Synthetic peptide within Human Histone H2B AA 1-100	1/1000	N/A[e]	Ubiquitous
SNRPA1	Anti-SNRPA1 antibody [EPR7557] (ab128937)	Synthetic peptide corresponding to residues in Human SNRPA1	1/100	N/A[e]	Ubiquitous

[a]Primary antibodies supplied by Abcam Inc (Cambridge, MA, USA)

[b]Exact immunogen sequence is proprietary

[c]Linker molecules supplied by Aligent (Santa Clara, CA, USA)

[d]Recommended by antibody supplier, Abcam Inc (Cambridge, MA, USA), and assessed by MK in the standardisation of IHC staining protocol

[e]No linker used

Amino acid (AA); Immunohistochemistry (IHC); Ovarian carcinoma (OC); Ovarian Tumour Tissue Analaysis (OTTA); Tissue microarray (TMA)

Supplementary table 2 OTTA studies participating in the study and ethics approval

**Uploaded separately**

Supplementary table 3 On-slide positive and negative tissue controls for GMNN and FOXJ1 IHC staining, arrayed on TMAs examined in the final scoring cohorts of 5503 and 7759 OC cases, respectively

Study[a]	GMNN			FOXJ1		
	n[b]	On-slide controls[c]		n[b]	On-slide controls[c]	
		Positive[d]	Negative		Positive[d]	Negative
AOV	14	W	J, K, P, R	98	N/A[e]	J, K, P, R, W
CAL	8	W	N/A	8	N/A[e]	W
CNI	18	A, B, C, D, E, F, H, L, M, Q, T, U, W	R, V	18	F, L, T	A, B, C, D, E, H, M, Q, R, U, V, W
DOV	8	L, Q, W	I, J, K, R, S	8	L	I, J, K, Q, R, S, W
DUK	0	N/A	N/A	0	N/A[e]	N/A[e]
HAW	48	A, D, W	J, K, P, R, S	48	N/A[e]	A, D, J, K, P, R, S, W
HOP	8	N/A	I, J, K, S	8	N/A[e]	I, J, K, S
LAX	76	N/A	N, O	76	O	N
POC	0	N/A	N/A	0	N/A[e]	N/A[e]
SEA	32	N/A	I, J, K, S	32	N/A[e]	I, J, K, S
TVA	68	Q, W	J, K, P	68	N/A[e]	J, K, P, Q, W
UKO	16	N/A	I, J, K, S	16	N/A[e]	I, J, K, S
VAN	64	D, F	G	64	F, G	D
AOC				16	N/A[e]	K
BAV				0	N/A[e]	N/A[e]
MAY				0	N/A[e]	N/A[e]

[a]OTTA studies participating in this project; Supplementary table 2 details their contribution

[b]Total number of control cores on all TMAs analysed, from each study; in cases where the number of cores does not match the listed tissues, some tissue cores were in replicates

[c]Studies that contributed multiple TMAs had on-slide controls on all TMAs

[d]A lack of data on GMNN expression analysed by IHC in these tissue types meant they could not be separated into low and high expressor positive controls

[e]No control tissue present

Breast (A); Breast carcinoma (B); Chronic lymphocytic leukemia (C); Colon (D); Endocervix (E); Endometrium (F); Fallopian tube (G); Glioblastoma (H); Heart (I); Immunohistochemistry (IHC); Kidney (J); Liver (K); Lung (L); Lymphoma (M); Ovarian fibroma (N); Ovarian serous cystadenoma (O); Pancreas (P); Placenta (Q); Prostate (R); Spleen (S); Stomach (T); Tissue microarray (TMA); Testicular tumour (U); Thyroid (V); Tonsil (W)

Supplementary table 4 Top 90 prognostic genes identified by NanoString analysis, ranked by their association with HGSC survival (n=3,769)

Ranking[a]	Gene	Abcam antibody[c]	Localisation	Staining[b]
1	TAP1	Yes	Cytoplasmic	Failed
2	CXCL9	Yes	Cytoplasmic	Failed
3	ZFHX4	No		
4	PTGER3	Yes	Membranous	Failed
5	CD38	Yes	Membranous	Good
6	TRIM27	No		
7	CXCL10	No		
8	FBN1	No		
9	TIMP3	Yes	Cytoplasmic/stromal	
10	SLAMF7	Yes	Membranous	
11	COL3A1	No		
12	IGHM	No		
13	SERPINE1	No		
14	CXCL11	No		
**15**	**GMNN**	**Yes**	**Nuclear**	**Good**
16	SVIL	No		
**17**	**SNRPA1**	**Yes**	**Nuclear**	**Good**
18	SPARC	Yes	Stromal	Failed
19	PCDH9	No		
20	MRPS27	No		
21	CD27	Yes	Membranous	
22	ASRGL1	No		
23	IGF1	No		
24	COL5A2	No		
25	CTSK	Yes	Cytoplasmic	
26	ENOX1	No		
27	FAP	No		
28	UCP2	No		
29	IDO1	Yes	Cytoplasmic	
30	FABP4	Yes	Cytoplasmic, nuclear	
31	PARP4	No		
32	DUSP1	Yes	Cytoplasmic	
33	DCN	Yes	Stromal	Failed
34	IGJ	Yes	Plasma cells	
35	ADH1B	No		
36	COL1A2	Yes	Stromal	
37	COL11A1	No		
38	MAP1LC3A	Yes	Cytoplasmic	
39	RASA1	Yes	Cytoplasmic	
40	PCK2	Yes	Mitochondrial	
41	GALNT6	No		
42	GJB1	Yes	Membranous	
43	LUM	Yes	Stromal	
44	TBC1D8B	No		
45	INHBA	Yes	Stromal	
46	ADAMDEC1	No		
47	NUAK1	No		

**48**	**FOXJ1**	**Yes**	**Nuclear**	**Good**
49	RBMS3	No		
50	CCL5	No		
51	HBB	No		
52	PD.L1	Yes	Cytoplasmic	
53	MPZL2	No		
54	FAM58A	No		
55	THBS2	No	Stromal	
56	FGF1	No		
57	CTLA4	Yes	Membranous	
58	C19orf12	No		
**59**	**HIST1H2BD**	**Yes**	**Nuclear**	**Good**
60	PLK2	No		
61	TMEM45A	No		
62	LOX	Yes	Cytoplasmic	
63	OLFML3	No		
64	CDK6	No		
65	SORL1	Yes	Cytoplasmic	
66	B4GALT5	No		
67	PDGFRB	Yes	Stromal	
68	SAC3D1	No		
69	ESD	No		
70	MAK	No		
71	SEMA4D	Yes	Cytoplasmic	
72	RB1	Yes	Nuclear	Good*
73	SACS	No		
74	ZC3H13	No		
75	HIST1H2BG	No		
**76**	**FEN1**	**Yes**	**Nuclear**	**Good**
77	GFRA1	No		
78	SUPT6H	No		
79	APC	Yes	Cytoplasmic	
80	APBB2	Yes	Cytoplasmic	
81	COL5A1	No		
82	TUBB6	No		
83	IGKC	Yes	Plasma cells	
84	ENPP1	Yes	Membranous	
85	LRRC15	No		
86	CRISPLD2	No		
87	IGF2	No		
88	CX3CR1	No		
89	APC	Yes	Cytoplasmic	
90	PDS5B	No		

[a]Genes ranked by prognostic significance

[b]Currently, not all genes have been assessed by IHC staining.

- High quality antibody either rabbit or mouse monoclonal suitable for IHC-P, judged based on pictures provided by manufacture
- [c] Manuscript in preparation

Supplementary table 5 Clinicopathological characteristics by histotype of the 5470 OC patients, with complete survival data, examined by GMNN expression

**Characteristic**	**HGSC**	**LGSC**	**MC**	**EC**	**CCC**
Number of cases, n (%)[a]	4185 (76.5)	160 (2.9)	174 (3.2)	518 (9.5)	433 (7.9)
Age at diagnosis, years					
Mean ± SD	60.4 ± 10.6	55.0 ± 12.3	53.2 ± 14.6	55.0 ± 11.1	55.5 ± 11.0
Median	61	56	53	54	55
Range	21-92	23-88	16-83	28-88	28-89
Stage, n (%)[b]					
FIGO I, II (localised)	802 (19.2)	51 (31.9)	147 (84.5)	442 (85.3)	344 (79.5)
FIGO III, IV (distant)	3383 (80.8)	109 (68.1)	27 (15.5)	76 (14.7)	89 (20.6)
Outcome[c]					
Alive, n (%)[b]	1259 (29.8)	66 (41.3)	119 (68.4)	371 (71.6)	263 (60.7)
Dead, n (%)[b]	2949 (70.2)	94 (58.8)	55 (31.6)	147 (28.4)	170 (39.3)
5-year survival, % ± SE	39.1 ± 0.8	59.4 ± 4.3	68.1 ± 4.1	81.6 ± 1.9	65.7 ± 2.5

[a]The proportion of cases in each histotypes is given as a percentage of the total patients examined
[b]The proportion of cases is given as a percentage of the total cases within each histotypes
[c]Final status of the patient, being alive or dead, at 10years, following enrollment in an OTTA

Clear cell ovarian carcinoma (CCC); Endometroid ovarian carcinoma (EC); High-grade serous ovarian carcinoma (HGSC); International Federation of Gynecology and Obstetrics (FIGO); Low-grade serous ovarian carcinoma (LGSC); Standard deviation (SD); Standard error (SE)

Supplementary table 6 Association of stratified FOXJ1 and GMNN protein expression and OS in HGSC, where cases were known to have not been treated with NACT (n=4440 and 4009 respectively)

**Marker**	**Expression**	**n[a]**	**5-yr survival (% ± SE)**	**HR (95% CI)[b]**	**p-value**
FOXJ1	0%	1269	36.5 ± 1.4	ref	**0.0003***
	5%	928	35.0 ± 1.6	1.03 (0.93-1.14)	
	10%-15%	881	41.6 ± 1.7	0.91 (0.82-1.01)	
	20%-45%	1018	43.5 ± 1.6	**0.86 (0.78-0.95)***	
	50%-100%	344	49.4 ± 2.9	**0.77 (0.66-0.90)***	
GMNN	0%	244	40.4 ± 3.3	ref	**0.003***
	5%	731	40.0 ± 1.8	1.10 (0.92-1.31)	
	10%-15%	1335	38.9 ± 1.3	1.08 (0.91-1.28)	
	20%-25%	922	40.3 ± 1.6	0.95 (0.79-1.13)	
	30%	178	36.9 ± 3.6	1.0 (0.80-1.28)	
	35%-100%	236	46.8 ± 3.3	0.81 (0.64-1.02)	

[a]The same cohort was assessed in univariate survival analysis
[b]HR adjusted for patient age, stage and OTTA study; Cox proportional regression modelling was used to calculate p-values and define significance. Statistically significant values shown in bold; * $p<0.05$

Confidence interval (CI); High-grade serous ovarian carcinoma (HGSC); Hazard ratio (HR); Overall survival (OS)
