# Supplementary material for: Increased FOXJ1 protein expression is associated with improved overall survival in high-grade serous ovarian carcinoma: an Ovarian Tumor Tissue Analysis Consortium Study
Source: Br J Cancer. 2022 Nov 2;128(1):137–47. doi: 10.1038/s41416-022-02014-y (PMC9814937; doi:10.1038/s41416-022-02014-y)
Supplement: Supplementary file 4 — Supplementary Table 2 [file 41416_2022_2014_MOESM4_ESM.pdf]

Increased FOXJ1 protein expression is associated with improved overall survival in high-grade serous ovarian carcinoma: an Ovarian Tumor Tissue Analysis Consortium Study

Supplementary table 2 OTTA studies participating in the study and ethics approval

OTTA study	Name	Location	Years	Ascertainment of patients and clinical data	Pathology data and review	Ethics committee	Informed consent	FOXJ1 cohort n (%) $^{a\ b}$	GMNN cohort n (%) $^{a\ b}$
AOC	Australian Ovarian Cancer Study	Australia	2002-2006	Treatment centers throughout Australia; cancer registries serving Queensland, South and West Australia; regular follow-up visits	Central review of pathology reports and histological slides by study pathologist	Peter MacCallum Cancer Centre Human Research Ethics Committee	Yes	207 (3.2)	199 (3.6)
AOV	Alberta Ovarian Tumor Types Study	Canada	1978-2010	Population-based Alberta Cancer Registry; annual updates are performed for vital statistics	Central review of pathology reports and histological slides by study pathologist	Alberta Health Services, Research Ethics	No / pathology material	416 (6.5)	63 (1.2)
BAV	Bavarian Ovarian Cancer Study	Germany	2002-2006	Gynaecologic Oncology Center at the Comprehensive Cancer Center Erlangen-Nuremberg	Central review of pathology reports and histological slides by study pathologist	Ethics Committee of the Friedrich-Alexander-University Erlangen-Nuremberg	Yes	214 (3.3)	192 (3.5)
BRZ	Brazil Gynecologic	Brazil	1987-2010	University Hospital of Ribeirao Preto	Pathology reports and histologic slides reviewed	Research Ethics Committee of Hospital das	No / pathology material	100 (1.6)	86 (1.6)

	Tumor Bank (BRZ) study			School of Medicine (HCRP), case series with prospective follow up	by HCRP gynaecologic pathologists	Clínicas of the Ribeirão Preto Medical School			
CAL	Calgary Serous Carcinoma Study	Canada	2003-2007	Hospital based retrospective observational study	Central review of pathology reports and histological slides by study pathologist	Conjoint Health Research Ethics Board	Yes	107 (1.7)	101 (1.9)
CNI	Spanish National Cancer Research Centre Ovarian Cancer Study	Spain	2006-2013	Hospitals in Madrid in Medical Oncology Divisions	Pathology information was obtained through medical chart review in the Medical Oncology units	Bioethics and Animal Welfare Committee of the Carlos III Health Institute	Yes	68 (1.1)	33 (0.6)
DOV	Diseases of the Ovary and their Evaluation	United States	2002-2009	Fred Hutchinson Cancer Research Center	Two stage review: Central review of pathology reports and histological slides by study pathologist. Histotype was then assigned by the pathologist through 2014 WHO diagnostic classification guidelines and re-reviewing the H&E stained slide of the primary tumour	Fred Hutchinson Cancer Research Center Institutional Review Board	Yes	1054 (16.4)	1042 (19.1)
DUK	Duke University Medical Center	United States	2008-2009	Duke University Medical Center, North Carolina	Stained histological slides reviewed centrally by study pathologist	Duke University Health System Institutional Review Board	Yes	39 (0.6)	42 (0.8)
HAW	Hawaii Ovarian Cancer Study	United States	1993-2008	Hawaii Tumor Registry and medical records	Central review of pathology reports and histological slides by study pathologist	University of Hawaii, Committee on Human Studies	Yes	122 (1.9)	122 (2.2)

HMC	(HM-Ciocc) HM Hospitales – Centro Integral Oncológico HM Clara Campa	Spain	2016-2021	Department of Gynecologycal Tumors at HM Hospitals	Central review of pathology reports and histological slides by study pathologist	Research and Ethics committee of HM hospital	Yes	70 (1.1)	69 (1.3)
HOP	Hormones and Ovarian Cancer PrEdiction	United States	2003-2009	Hospital registries and active surveillance of medical practices in Western PA, North-eastern OH, and Western NY	Pathology information was obtained through medical chart review in the Medical Oncology units	University of Pittsburgh Insititutional Review Board and Roswell Park Cancer Institute Insititutional Review Board	Yes	36 (0.6)	37 (0.7)
LAX	Women's Cancer Research Program - Cedars-Sinai Medical Center	United States	1989-present	Women's Cancer Program Biorepository	Central review of pathology reports and histological slides by study pathologist	Institutional Review Board 3 of Cedars-Sinai Medical Center	Yes	147 (2.3)	139 (2.5)
MAY	Mayo Clinic Ovarian Cancer Study	United States	2000-2013	Mayo Clinic medical records and death certificates	Central review of pathology reports and histological slides by study pathologist	Institutional Review Board of Mayo Clinic	Yes	1034 (16.1)	584 (10.7)
NCT	Natl. Centre of Tumor Diseases and the Dept. of Pathology, Heidelberg	Germany	2004-2016	Natl. Centre of Tumor Diseases and the Dept. of Pathology, Heidelberg	Pathology reports and histologic slides reviewed by gynaecologic pathologist	Ethics-Committee of the Medical Faculty at the University of Heidelberg	Yes	40 (0.6)	41 (0.8)
POC	Polish Ovarian Cancer Study	Poland	2000-2003	Hospital records and cancer registries serving Warsaw and Lodz	Stained histological slides reviewed centrally by study pathologist	Bioethical Committee of Pomeranian Medical University	Yes	83 (1.3)	80 (1.5)

SEA	Study of Epidemiology and Risk Factors in Cancer Heredity	United Kingdom	1998-present	Eastern Region Cancer Intelligence Unit, West Midlands Cancer Intelligence Unit, and multiple cancer networks	Central review of pathology reports and histological slides by study pathologist	Cambridgeshire 4 Research Ethics Committee	Yes	549 (8.5)	521 (9.5)
TUE	Tuebingen University Women's Hospital (TUE) study	Germany	1999-2008	Department of Obstetrics and Gynaecology, Eberhard Karls Universitats Tübingen, Tübingen Germany	Pathology reports and histologic slides reviewed by gynaecologic pathologist	Ethics-Committee at the Medical Faculty and at the University Hospital of Tübingen	Yes	388 (6.0)	366 (6.7)
TVA	Ovarian Cancer in Alberta	Canada	2004-2012	Alberta Cancer Registry and Provincial Cancer Treatment Centers	Central review of pathology reports and histological slides by study pathologist	University of Calgary, Conjoint Health Research Ethics Committee	Yes	138 (2.1)	136 (2.5)

UKO	United Kingdom Ovarian Cancer Population study	United Kingdom	2006-2010	Ten major Gynaecologic Oncology NHS centers in England, Wales and Northern Ireland; cancer registries; NHS Information Centre for Health and Social Care (England and Wales) and Central Services Agency (Northern Ireland)	Central review of pathology reports by gynaecologic oncologist	National Health Service Central Office for Research Ethics Committees (COREC) and The Joint University College London/University College London Hospital Committee on the Ethics of Human Research (Committee A)	Yes	102 (1.6)	102 (1.9)
VAN	Vancouver Ovarian Cancer Study	Canada	1984-2000	Ovarian Cancer Registry serving British Columbia and the Cheryl Brown Outcomes Unit	Central review of pathology reports and histological slides by study pathologist	University of British Columbia - British Columbia Cancer Agency Research Ethics Board	Some cases yes and some cases no / pathology material	1320 (20.5)	1327 (24.3)
WMH	WestMead Hospital	Australia	1992-2014	The Crown Princess Mary Cancer Centre and affiliated hospitals	Pathology reports and diagnostic slides reviewed by panel of gynaecologic pathologists	Western Sydney Local Health District, Human Research Ethics Committee	Yes	200 (3.1)	189 (3.5)
					**Total cohort**			6434 (100.0)	5470 (100.0)

[a] Sample sizes reflect ovarian carcinoma cases that have met the inclusion criteria for this study
[b] The proportion of cases is given as a percentage of the total cases assessed for each marker
