# Supplementary material for: Increased FOXJ1 protein expression is associated with improved overall survival in high-grade serous ovarian carcinoma: an Ovarian Tumor Tissue Analysis Consortium Study
Source: Br J Cancer. 2022 Nov 2;128(1):137–47. doi: 10.1038/s41416-022-02014-y (PMC9814937; doi:10.1038/s41416-022-02014-y)
Supplement: Supplementary file 5 — AOCS Study Group [file 41416_2022_2014_MOESM5_ESM.pdf]

AOCS STUDY GROUP
Management Group: D Bowtell[1,3,4,5,6], G Chenevix-Trench[2], A Green[2], P Webb[2], A DeFazio[7,8,9], D Gertig[10]

Project and Data Managers: N Traficante[1,4], S Fereday[1,4], S Moore[2], J Hung[7], K Harrap[2], T Sadkowsky[2], N Pandeya[2]

Research Nurses and Assistants:
M Malt[2], A Mellon[11], R Robertson[11], T Vanden Bergh[12], M Jones[12], P Mackenzie[12], J Maidens[13], K Nattress[14], YE Chiew[7], A Stenlake[9], H Sullivan[9], B Alexander[2], P Ashover[2], S Brown[2], T Corrish[2], L Green[2], L Jackman[2], K Ferguson[2], K Martin[2], A Martyn[2], B Ranieri[2], J White[15], V Jayde[16], P Mamers[17], L Bowes[1], L Galletta[1], D Giles[1], J Hendley[1], K Alsop[1], T Schmidt[18], H Shirley[18], C Ball[19], C Young[19], S Viduka[18], Hoa Tran[18], Sanela Bilic[18], Lydia Glavinas[18], Julia Brooks[20]

Clinical and Scientific Collaborators:
R Stuart-Harris[21], F Kirsten[22], J Rutovitz[23], P Clingan[24], A Glasgow[24], A Proietto[11], S Braye[11], G Otton[11], J Shannon[25], T Bonaventura[26], J Stewart[26], S Begbie[27] M Friedlander[28] D Bell[13], S Baron-Hay[13], A Ferrier[13] (*dec.*), G Gard[13], D Nevell[13], N Pavlakis[13], S Valmadre[13], B Young[13], C Camaris[12], R Crouch[12], L Edwards[12], N Hacker[12], D Marsden[12], G Robertson[12], P Beale[14], J Beith[14], J Carter[14], C Dalrymple[14], R Houghton[14], P Russell[14], M Links[29], J Grygiel[30], J Hill[31], A Brand[8,32], K Byth[32], R Jaworski[33], P Harnett[8,32], R Sharma[8,33], G Wain[32], B Ward[34], D Papadimos[34], A Crandon[35], M Cummings[35], K Horwood[35], A Obermair[35], L Perrin[35], D Wyld[35], J Nicklin[35,36], M Davy[15], MK Oehler[15], C Hall[15], T Dodd[15], T Healy[37], K Pittman[37,] D Henderson[37], J Miller[39], J Pierdes[39], P Blomfield[16], D Challis[16], R McIntosh[16], A Parker[16], B Brown[40], R Rome[40], D Allen[41], P Grant[41], S Hyde[41], R Laurie[41], M Robbie[41,] D Healy[17], T Jobling[17], T Manolitsas[17], J McNealage[17], P Rogers[17], B Susil[17], E Sumithran[17], I Simpson[17], K Phillips[1], D Rischin[1], S Fox[1], D Johnson[1], S Lade[1], M Loughrey[1], N O'Callaghan[1], W Murray[1], P Waring[3], V Billson[42], J Pyman[42], D Neesham[42], M Quinn[42], C Underhill[43], R Bell[44], LF Ng[45], R Blum[46], V Ganju[47], I Hammond[19], Y Leung[19], A McCartney[19] (*dec.*), M Buck[48,] I Haviv[49], D Purdie[2], D Whiteman[2], N Zeps[18]

[1]Peter MacCallum Cancer Centre, Melbourne, Victoria, 3000, Australia.

[2]QIMR Berghofer Medical Research Institute, Brisbane, Queensland, 4006, Australia.

[3]Department of Pathology, University of Melbourne, Parkville, Victoria, 3052, Australia.

[4]Sir Peter MacCallum Cancer Centre Department of Oncology, University of Melbourne, Parkville, Victoria, 3052, Australia.

[5]Department of Biochemistry and Molecular Biology, University of Melbourne, Parkville, Victoria, 3052, Australia.

[6]Ovarian Cancer Action Research Centre, Department of Surgery and Cancer, Imperial College London, London, England, W12 0HS, UK.

[7]Centre for Cancer Research, The Westmead Institute for Medical Research, Sydney, New South Wales, 2145, Australia

[8]The University of Sydney, Sydney, New South Wales, 2006, Australia.

[9]Department of Gynaecological Oncology, Westmead Hospital, Sydney, New South Wales, 2145, Australia.

[10]Melbourne School of Population and Global Health, University of Melbourne, Parkville, Victoria, 3052, Australia.

[11]John Hunter Hospital, Lookout Road, New Lambton, New South Wales, 2305, Australia

[12]Royal Hospital for Women, Barker Street, Randwick, New South Wales, 2031, Australia

[13]Royal North Shore Hospital, Reserve Road, St Leonards, New South Wales, 2065, Australia

[14]Royal Prince Alfred Hospital, Missenden Road, Camperdown, New South Wales, 2050, Australia

[15]Royal Adelaide Hospital, North Terrace, Adelaide, South Australia, 5000, Australia

[16]Royal Hobart Hospital, 48 Liverpool St, Hobart, Tasmania, 7000, Australia

[17] Monash Medical Centre, 246 Clayton Rd, Clayton, Victoria, 3168, Australia

[18]Western Australian Research Tissue Network (WARTN), St John of God Pathology, 23 Walters Drive, Osborne Park, Western Australia, 6017, Australia

[19]Women and Infant's Research Foundation, King Edward Memorial Hospital, 374 Bagot Road, Subiaco, Western Australia, 6008, Australia

[20]St John of God Hospital, 12 Salvado Rd, Subiaco, Western Australia, 6008, Australia

[21]Canberra Hospital, Yamba Drive, Garran, Australian Capitol Territory, 2605, Australia

[22]Bankstown Cancer Centre, Bankstown Hospital, 70 Eldridge Road, Bankstown, New South Wales, 2200, Australia

[23]Northern Haematology & Oncology Group, Integrated Cancer Centre, 185 Fox Valley Road, Wahroonga, New South Wales, 2076, Australia

[24]Illawarra Shoalhaven Local Health District, Wollongong Hospital, Level 4 Lawson House, Wollongong, New South Wales, 2500, Australia

[25]Nepean Hospital, Derby Street, Kingswood, New South Wales, 2747, Australia

[26]Newcastle Mater Misericordiae Hospital, Edith Street, Waratah, New South Wales, 2298, Australia

[27]Port Macquarie Base Hospital, Wrights Road, Port Macquarie, New South Wales, 2444, Australia

[28]Prince of Wales Clinical School, University of New South Wales, New South Wales, 2031, Australia

[29]St George Hospital, Gray Street, Kogarah, New South Wales, 2217, Australia

[30]St Vincent's Hospital, 390 Victoria Street, Darlinghurst, New South Wales, 2010, Australia

[31]Wagga Wagga Base Hospital, Docker St, Wagga Wagga, New South Wales, 2650, Australia

[32]Crown Princess Mary Cancer Centre, Westmead Hospital, Westmead, Sydney, New South Wales, 2145, Australia.

[33]Department of Pathology, Westmead Clinical School, Westmead Hospital, The University of Sydney, New South Wales, 2006, Australia

[34]Mater Misericordiae Hospital, Raymond Terrace, South Brisbane, Queensland, 4101, Australia

[35]The Royal Brisbane and Women's Hospital, Butterfield Street, Herston, Queensland, 4006, Australia

[36] Wesley Hospital, 451 Coronation Drive, Auchenflower, Queensland, 4066, Australia

[37]Burnside Hospital, 120 Kensington Road, Toorak Gardens, South Australia, 5065, Australia

[38]Flinders Medical Centre, Flinders Drive, Bedford Park, South Australia, 5042, Australia

[39]Queen Elizabeth Hospital, 28 Woodville Road, Woodville South, South Australia, 5011, Australia

[40]Freemasons Hospital, 20 Victoria Parade, East Melbourne, Victoria, 3002, Australia

[41]Mercy Hospital for Women, 163 Studley Road, Heidelberg, Victoria, 3084, Australia

[42]The Royal Women's Hospital, Parkville, Victoria, 3052, Australia

[43]Border Medical Oncology, Wodonga, Victoria, 3690, Australia

[44]Andrew Love Cancer Centre, 70 Swanston Street, Geelong, Victoria, 3220, Australia

[45]Ballarat Base Hospital, Drummond Street North, Ballarat, Victoria, 3350, Australia

[46]Bendigo Health Care Group, 62 Lucan Street, Bendigo, Victoria, 3550, Australia

[47]Peninsula Health, 2 Hastings Road, Frankston, Victoria, 3199, Australia

[48]Mount Hospital, 150 Mounts Bay Road, Perth, Western Australia 6000, Australia

[49]Faculty of Medicine, Bar-Ilan University, 8 Henrietta Szold St, Safed, Israel
